# A network analysis of the propagation of evidence regarding the effectiveness of fat-controlled diets in the secondary prevention of coronary heart disease (CHD): Selective citation in reviews

**DOI:** 10.1371/journal.pone.0197716

**Published:** 2018-05-24

**Authors:** Rhodri Ivor Leng

**Affiliations:** Department of Science, Technology and Innovation Studies, School of Social and Political Science, University of Edinburgh, Edinburgh, United Kingdom; Public Library of Science, UNITED KINGDOM

## Abstract

**Objective:**

To examine how the first randomised controlled trials (RCTs) evaluating the efficacy of cholesterol-lowering diets in the secondary prevention of coronary heart disease were interpreted in reviews of the literature prior to the National Institutes of Health consensus conference in 1984.

**Design:**

Claim-specific citation network analysis was used to study the network of citations between reviews and RCTs over a defined period (1969–1984). RCTs were identified and classified according to whether their conclusions supported or opposed the use of dietary fat modification/restriction in the secondary prevention of coronary heart disease. Each review published in this period that cited any of the RCTs was classified as supportive, neutral, or unsupportive to the use of dietary fat modification based on a quotation analysis of its evaluation of the findings of these RCTs. Citation bias and underutilisation were detected by applying a comparative density measure, in-degree centrality, and out-degree in a series of sub-graph analyses.

**Results:**

In total, 66 unique publications were identified (four RCTs—one supportive, three unsupportive; 62 reviews—28 supportive, 17 neutral, 17 unsupportive). On average, supportive reviews underutilised the available RCTs to a greater degree than other reviews. Amongst the supportive group, citation bias was common—23 (82%) reviews cited only the one RCT that was supportive.

**Conclusion:**

Most reviews that disseminated a supportive evaluation of the results of RCTs in the context of secondary prevention cited only data that supported this position.

## Introduction

In 1961, the *American Heart Association* published guidelines recommending that people who have had one or more atherosclerotic heart attacks should reduce their intake of saturated fatty acids and modestly increase that of polyunsaturated fatty acids [1]. The rationale was an early version of the *diet–heart hypothesis*, that dietary intake of saturated fatty acids, by increasing total serum cholesterol, contributes to coronary heart disease (CHD) via the development of atherosclerosis, while polyunsaturated fatty acids decrease serum cholesterol and lower the risk of CHD [2]. In the 1950s, this theory was supported by: (i) epidemiological evidence of an association between fat intake, particularly saturated fatty acids intake, and raised serum cholesterol levels; (ii) an association between serum cholesterol and coronary incidence and mortality; (iii) short-term metabolic ward trials that suggested that saturated fatty acids increased serum cholesterol while polyunsaturated fatty acids appeared to modestly lower serum cholesterol [3].

However, some scientists warned of the dangers of mistaking correlation for causation, particularly in population studies [4] and in a multifactorial disease such as CHD [5, 6]. By the time that the guidelines were issued, no randomised controlled trial (RCT) had tested the link between the consumption of different types of fat and coronary risk in secondary prevention (i.e., after a clinical manifestation of CHD), but, in the 1960s, five RCTs examined this. The *Los Angeles Veterans Administration Diet Study* [7] combined secondary prevention and primary prevention (i.e., before the development of CHD), but was generally regarded as relevant only to primary prevention due to the way the authors stratified their data and presented their findings. Four other trials specifically examined the effect of a modified diet in the secondary prevention of CHD in patients who had experienced a recent myocardial infarction: the *Research Committee Low-Fat Diet Study* [8] examined total fat restriction, while the *Rose Corn Oil Study* [9], the *Oslo Diet-Heart Study* [10], and the *Medical Research Council’s Soya-bean Oil Study* [11] examined the impact of restricting saturated fatty acids and increasing polyunsaturated fatty acid intake. These were the only published secondary prevention RCTs until the partial publication of the *Sydney Diet–Heart Study* [12] in 1978, which only published results concerning all-cause mortality. The full results of that trial on cardiovascular disease (CVD) and CHD mortality were recently recovered and re-analysed by Ramsden *et al*. [13], who found a significant *harmful* effect of replacing saturated fatty acids with polyunsaturated fatty acids on all-cause mortality, CVD mortality, and CHD mortality.

The four RCTs published in 1965 [8, 9], 1966 [10], and 1968 [11] had similar methodologies, and were published in major journals, but produced discrepant findings (S1–S4 Tables). The *Oslo* study, published in 1966, found significantly lower levels of fatal reinfarction, non-fatal reinfarction, and angina pectoris in the intervention group, but found no significant relationship between diet and sudden death or all-cause mortality [10]. The other three studies all returned equivocal or negative results regarding reinfarction, coronary mortality, cardiovascular events, and all-cause mortality. The author of the *Oslo* study regarded his results as supporting dietary fat modification in secondary prevention, while the authors of the other studies regarded their results as evidence against such treatments.

In December 1984, the National Institutes of Health (NIH) gathered a panel of experts to resolve whether the link between raised serum cholesterol and heart attack was causal, and under what circumstances dietary or drug therapy should be initiated [14]. After reviewing the available evidence, the panel concluded that an elevated serum cholesterol level is a cause of CHD and that lowering it is protective. To lower serum levels of cholesterol, the panel recommended that all men, women, and children over 2 years of age should eat a diet consisting of no more than 30% fat, should reduce their intake of saturated fatty acids to less than 10% of total calories, and should increase their intake of polyunsaturated fatty acids to 10% of total calories. Specifically, on secondary prevention, the panel concluded:

“Studies are available that indicate a beneficial effect of treating high cholesterol levels in individuals with preexisting clinical disease (secondary intervention) … It is encouraging that the progression of established lesions may be retarded by appropriate **dietary** and drug therapy” (p.2084)

The conference was widely regarded as bringing a (temporary) end to the controversy over both the relationship of cholesterol with CHD and the relationship between dietary fats and CHD [15].

Yet, by 1984, the four RCTs discussed previously remained the only available RCTs with data on the effect of dietary modification on coronary mortality and morbidity in secondary prevention. However, the results of these trials were conflicting–an evaluation supported both by the directors of each study and by recent meta-analyses [16–19]. According to Harcombe *et al*. [16], the NIH advice was *not* supported by the available RCTs, neither in primary or secondary prevention. As can be seen in the risk-ratio analysis in the supplement (S4 Table), the evidence from these RCTs available prior to 1984 leads to no clear conclusion as to whether dietary modification is an appropriate treatment in secondary prevention. Recent meta-analyses also agree that, when the findings of these four trials are considered together, no significant difference exists on the “hard” clinical end-points–the incidence of myocardial infarction, fatal CHD, and all-cause mortality–between the intervention and control [16–19].

The present study examines how these four trials were utilised in the literature and their impact on published scientific opinion regarding the case for dietary fat modification/restriction in the secondary prevention of CHD, prior to the NIH consensus conference. By using a modified version of *claim-specific* citation network analysis, a methodology developed by Greenberg [20], it maps and evaluates the network of review papers that cited these RCTs over a defined period (1969–1984); the date by which all relevant RCT data would have been available to reviewers to the start of the NIH conference. The rationale for examining how these studies were cited is the claim that the diet–heart controversy stems from selective citation [21, 22]. In 1992, Uffe Ravnskov, in one of the first published demonstrations of citation bias, examined 22 cholesterol-lowering trials and found that supportive trials were cited almost six times as often as others [21]. In three highly-cited reviews associated with dietary policy [23–25], he went on to document citation bias and quotation bias, which describes the erroneous or selective interpretation of experimental findings [22]. Finally, in a response to an article in the *BMJ* in 2003, he claimed that belief in the diet–heart hypothesis was kept alive by selective citation [26].

The present study tested these claims by examining how dietary RCT results were cited in reviews during the period leading up the publication of dietary guidelines and closure of controversy, following Greenberg’s [20] finding that reviews tend to be the major propagators of information regarding the findings of primary studies. To do so, it examines two outcomes of selective citation: *citation bias*–the selective citing of data supporting a particular outcome [20]; and *research underutilisation*–when authors fail to reference all available data relevant to a specific claim. These are outcomes of sub-optimal citation practices that distort the evidence base by inflating the influence of some studies while under-representing others [20, 27]. To detect these factors, network analysis was used to document citation bias and underutilisation by mapping the citation links between RCTs and reviews that were themselves classified by quotation analysis.

## Materials and methods

### Study selection

To be included, a trial had to be a unifactorial RCT that: (i) was published before 1984; (ii) examined the effect of dietary fat restriction (<30% of total calories derived from dietary fat) OR dietary fat modification (replacing saturated fatty acids with polyunsaturated fatty acids) on coronary benefit/risk in the secondary prevention of CHD; (iii) published data on sample numbers AND serum cholesterol changes AND mortality and morbidity figures regarding incidence of myocardial infarction, coronary mortality (including myocardial infarction and sudden death), and all-cause mortality; (iv) included *only* patients with established CHD (myocardial infarction, angina pectoris); (v) a minimum intervention and follow-up period of 12 months.

RCTs were identified via a literature search on *Web of Science* via the ‘all database’ function with the following Boolean string (“dietary fat” OR “fatty acids” OR “saturated fat” OR “low fat diet” OR “cholesterol lowering diet” OR “modified fat diet” OR “restricted fat diet” OR “corn oil” OR “soya bean oil” OR “unsaturated fat” OR “linoleic acid”) AND (“coronary heart disease” OR “ischaemic heart disease” OR “myocardial infarction” OR “cardiovascular disease” OR “atherosclerotic heart disease”) AND (“trial” OR “controlled trial” OR “randomised controlled trial” OR “intervention trial” OR “clinical trial”), limited to publications between 1950 and 1984. This returned 244 publications across four databases in March 2017 (*MEDLINE*, *Web of Science Core Collection*, *BIOSIS Citation Index*, *FSTA® - the Food Science Resource*). An additional examination of trials identified by recent systematic reviews and meta-analyses was also conducted to ensure all relevant studies were identified [16–19]. After screening publication titles and abstracts, 19 trials were identified as potentially relevant. After reading these, four RCTs were identified that met the inclusion criteria, all of which were published in the decade after the publication of the *American Heart Association* guidelines. The findings of these four RCTs were published in seven papers: the results of the *Oslo Trial* were published in four journals [10, 28–30]. These were merged, reducing the primary source studies to four. The results of these trials are included in the analysis in the supplement (S1–S4 Tables).

One trial, the *Los Angeles Veterans Administration Diet Study* [7], was omitted due to the inability to assess data on the effectiveness of dietary modification in secondary prevention due to the way the authors stratified their data and reported their findings. This was further justified by the prevailing opinion of the scientific community as expressed in reviews, which appeared to regard it relevant only to primary prevention, and its exclusion from meta-analyses covering secondary prevention specifically [18]. Another trial, the *Sydney Diet–Heart Study* [12], was omitted because only results on all-cause mortality were reported within the time frame and because it remained uncited in the literature by reviews until 1985 [30]. The *Finnish Mental Hospital Study* [31], a commonly-cited study in this period, was excluded on the basis that it was not an RCT; it was a “cross-over” trial in two mental hospitals and combined secondary and primary prevention. Further, it was not a standard cross-over trial: the patient groups were not the same in the diet arm and the control arm, no blinding was mentioned, and investigators re-coded deaths post-hoc. Bierenbaum et al.’s [32] *St Vincent’s Hospital Study* was excluded because of the lack of appropriate randomisation due to the assigning of a control group post-hoc that had “similar characteristics” to the intervention. Five trials were excluded on the basis of being primary prevention trials [33–35] and/or multifactorial [36–37], and six early secondary prevention trials were excluded due to a lack of appropriate randomisation and/or control [38–43].

Review papers, published in academic journals in English between 1969 and 1984 and which cited one or more of the studies, were identified by searching *Scopus* and *Web of Science* using indexed citation links associated with the relevant RCTs. To be included, a paper had to be a review or an extended editorial (with at least one citation) published in an academic journal within which the coronary mortality and morbidity results of the identified RCTs were discussed. During this period, only narrative reviews covered this topic, and none described a systematic search strategy. Excluded from analysis were letters, notes, book chapters, other primary articles, any paper not published in an academic journal, and any review that cited the RCTs without discussing mortality and morbidity. The search in *Scopus* returned 110 citing articles, of which 30 met the inclusion criteria. The search on *Web of Science* returned 215 citing articles, from which an additional 32 reviews were recovered that met the inclusion criteria. In total, 62 citing reviews were included in the analysis [44–105].

### Classification of papers

The RCTs were classified using ‘quotation analysis’ [20, 22, 27] according to whether the findings were evidence *for* or *against* the use of dietary fat modification/restriction in the secondary prevention of CHD. Quotations from the papers were collected to enable papers to be classified by the authors’ interpretation of their findings and these are reproduced in the supplement (S5 Table). For example, the authors of the *Rose Corn Oil Study*, an unsupportive trial, concluded that “under the circumstances of this trial corn oil cannot be recommended in the treatment of ischaemic heart disease” (p. 1533). By contrast, the author of the *Oslo Study*, the only supportive trial, concluded that the observed “reduction of the serum cholesterol level associated with a reduced CHD relapse rate strongly suggests a cause and effect relationship” (p. 79).

Reviews were similarly classified by quotation analysis by the authors’ evaluation of the RCT evidence. Quotations were identified either in the passage in which RCTs were cited or were stated explicitly in a conclusion, and these are reproduced in the supplement (S5 Table). Reviews were then classified into one of three categories: *supportive* reviews contained statements implying that the available trial evidence supported dietary intervention; *neutral* reviews contained statements highlighting the conflicting nature of the trial results and contained no statement either supporting or opposing dietary intervention; *unsupportive* reviews contained statements implying that the available trial evidence did not support the use of dietary intervention.

### Network data

Each paper was given an identifier code (V*n*). All authors, title of paper, year of publication, paper type (RCT or review), and classification were recorded to identify each vertex in a ‘node list’ format (S6 Table). This attribute information was used to examine the relationship between classification variables and citations behaviours. The citations *to* (incoming edges) and *from* (outgoing edges) all papers were recorded. The bibliographies of each review were screened to ensure that all citations to RCTs were identified within this sample. Citation information on the relationship between papers was recorded in an ‘edge list’ format in an Excel spreadsheet (S7 Table). This describes a network as a set of vertices (papers) connected by ‘edges’ (citation links). From these data, a graph was constructed and analysed using *R*’s ‘statnet’ package [106, 107] and visualised in *Gephi* [108]. Citation information was converted into an adjacency matrix that records edges (citations) between vertices (papers):

$$G_{ij}=\left\{ \begin{array}{l} 1\;if\;there\;is\;an\;edge\;between\;vertices\;i\;and\;j\\ 0\;otherwise\; \end{array}\right.$$

A graph [G] was constructed of the vertex set [V] and edge set [E]:

G=(V,E)

The vertices were coloured by classification and sized by in-degree, labelled by number assigned in the bibliography of this paper, and visualised initially using *Gephi’s* ‘ForceAtlas 2’ algorithm [108, 109] and later manually realigned in sub-graphs.

Two outcome measures–*citation bias* and *research underutilisation*–were used. Following Greenberg [20], *citation bias* refers to the act of citing only evidence of a particular classification. For example, if paper A references only supportive studies and no contradictory studies then it displays citation bias. Citation bias only describes the most extreme cases of evidence selection, but any underutilisation may create distortions in the literature–i.e., if reviews fail to use all relevant evidence even if a clear preference for a particular outcome does not influence selection. To detect these, the unit of analysis is the citation pathway, and the occupied citation pathway (an edge linking two vertices) is compared to all possible citation pathways between reviews and RCTs. Three measures are used to measure citation bias and underutilisation–a novel modified density measure, in-degree centrality, and out-degree.

The network in this study is an example of a *directed acyclic network* [110] because papers can only cite previously published papers and can never cite future literature or cite themselves. To know the *density*, the proportion of potential edges present, a measure of network connectivity is needed. Generally, if P is a set of primary studies, S a set of reviews, and E the number of edges from S to P, then

$$D=\frac{E}{\left|S|x|P\right|}$$

captures the number of times that a set of reviews cites a set of RCTs as a proportion of the maximum possible number of citations, allowing comparison between different classifications of review. Thus, this equation can be used to understand divergent utilisation of primary data.

*In-degree* reflects how often a study has been cited. The *in-degree* of a vertex is the number of edges from other vertices to that vertex, and, for a vertex *i*, is given by:

$$k_{i}^{in}=\sum_{j=1}^{n}G_{ij}$$

*Out-degree* reflects the number of references *from* a particular review. The out-degree of a vertex is the number of edges from that vertex to other vertices, given by:

$$k_{j}^{out}=\sum_{i=1}^{n}G_{ij}$$

To establish citation bias, the network was divided into three sub-graphs of the RCTs with reviews of each classification (supportive, neutral, unsupportive) and *only* edges from reviews to RCTs. The vertices that cite only RCTs of a particular classification were then counted–providing an exact account of citation bias and research utilisation.

Finally, a Pearson’s chi square test was performed on the distribution of citations to these four RCTs between the three classifications of review to establish whether the difference in citation behaviour is significant or simply the product of chance.

## Results

Of the four RCTs, one was supportive of dietary intervention [10] in the context of secondary prevention, while three were not [8, 9, 11]. Between 1969 and 1984, these were cited in 62 reviews, 28 of which were supportive of dietary intervention [78–105], 17 were unsupportive [61–77], and 17 ‘neutral’ reviews came to no clear conclusion on this issue [44–60]. Of these, 28 were published in the first 7 years (12 supportive; 12 neutral; four unsupportive), and 34 in the next 8 years (16 supportive; five neutral; 13 unsupportive).

**Fig 1:**
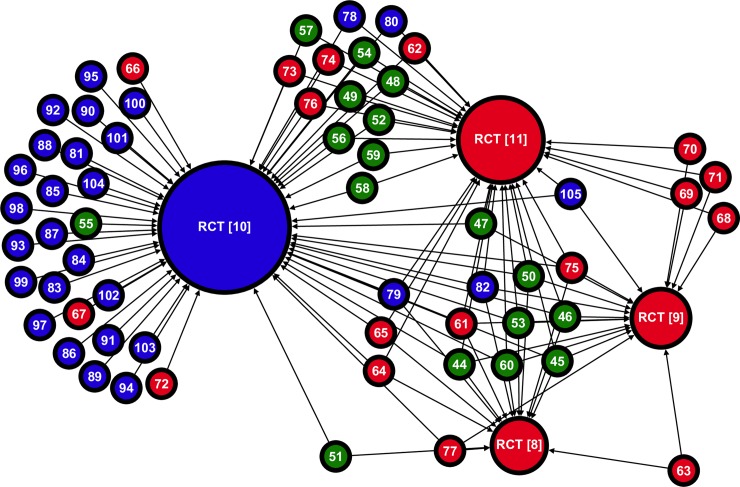
Citation network mapping the citations from reviews to RCTs testing dietary fat modification in the secondary prevention of CHD (*n* = 66; *m* = 121). Citation network graph with four RCTs connected to 62 citing reviews by a total of 121 citations. Each vertex represents a paper and the integer label corresponds to a paper in this paper’s bibliography. The colour of a vertex corresponds to a study’s classification (blue is supportive, green is neutral, and red is unsupportive). Vertex size is proportional to in-degree (total number of incoming citations). Each edge corresponds to a specific citation link and the direction of the edge is established by the direction of the arrow head. Visualised with Gephi’s ‘Force Atlas 2’ algorithm [109].

Fig 1 shows the citation network. Of the four RCTs, the most cited was the *Oslo* study with 57 citations. This was the only one of the four RCTs that was supportive of dietary intervention, but the other three (unsupportive) RCTs together attracted a similar number of citations (32 to *MRC Soya-bean*, 15 to *Rose Corn*, 17 to *Research Committee Low-fat*). Here, citation bias, particularly by supportive reviews, is signified by the many studies that cite *only* the *Oslo* study. Clearly, the distribution of citations to these studies is uneven. A high proportion of the reviews (27 of 62; 43%) cited only one of the RCTs, 20 cited two of them, six cited three and only nine cited all four. Nevertheless, taking the three unsupportive trials together, supportive trials are cited 57 times and unsupportive trials 64 times. Overall the graph has a density *D* of 0.49 –indicating only 49% of the possible citations exist between reviews and these RCTs.

To create an exact account of the utilisation of the primary RCT data by reviews, three sub-graphs were constructed including *only* reviews of a particular classification (supportive, neutral, unsupportive) and *only* edges from reviews to RCTs.

### Neutral reviews

**Fig 2:**
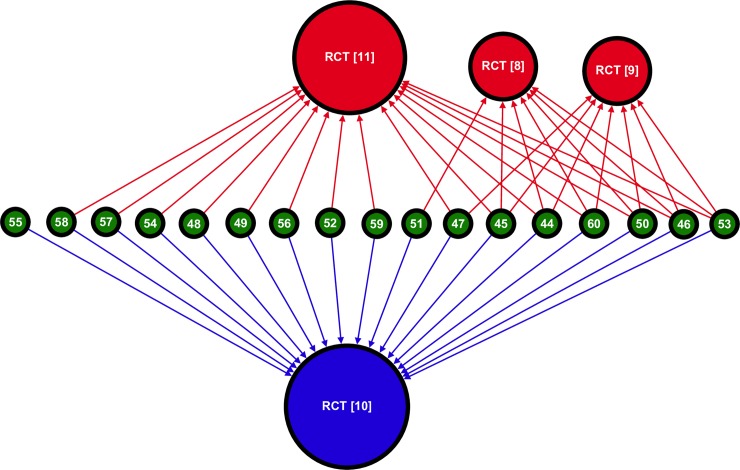
Sub-graph mapping the citations from neutral reviews to RCTs testing cholesterol lowering diets in the secondary prevention of CHD (*n* = 21; *m =* 46). This graph is composed of four RCTs connected to 17 citing reviews by a total of 46 citations. Each vertex represents a paper and the integer label corresponds to a paper in this paper’s bibliography. The colour of a vertex corresponds to a study’s classification (blue is supportive, green is neutral, and red is unsupportive). Vertex size is proportional to in-degree (total number of incoming citations). Each edge corresponds to a specific citation link, the direction of the edge is established by the direction of the arrow head, and the colour of an edge indicates the classification of the cited article. This was visualised manually in Gephi [108] by lining up reviews in the centre, placing supportive the RCT below and the unsupportive RCTs on top.

Of the neutral reviews (Fig 2), one cites only supportive data, which represent the only case of citation bias in this group. Overall the density of this sub-graph (*D* = 0.68) is higher than that of the network as a whole, indicating that reviews which cited more of the available primary literature were more likely to come to no clear conclusion than the average. While the *Oslo Study* received most citations (17), its influence is nearly rivalled by the *MRC Soya-bean Study*, which received 15; the *Rose Corn Oil Study* and the *Research Committee Low-fat Study* were each cited seven times. Six of the reviews cited all four RCTs and only one cited only one of them. Thus, compared to the whole network, neutral reviews were more likely to fully utilise the available RCT evidence and less likely to cite only one RCT.

### Unsupportive reviews

**Fig 3:**
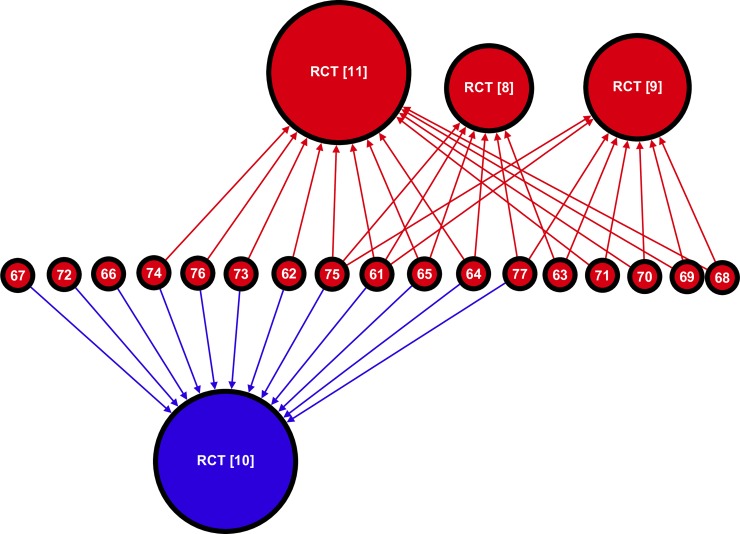
Sub-graph mapping the citations from unsupportive reviews to RCTs testing cholesterol lowering diets in the secondary prevention of CHD (*n* = 21; *m =* 38). This graph is composed of four RCTs connected to 17 reviews by a total of 38 citations. Each vertex represents a paper and the integer label corresponds to a paper in the reference list. The colour of a vertex corresponds to a study’s classification (blue is supportive and red is unsupportive). Vertex size is proportional to in-degree (total number of incoming citations). Each edge corresponds to a specific citation link, the direction of the edge is established by the direction of the arrow head, and the colour of an edge indicates the classification of the cited article.

The sub-graph of unsupportive reviews (Fig 3) had a density of 0.56. The *Oslo* study and the *MRC* study were each cited 12 times by unsupportive reviews, the *Rose* study six times and the *Low-fat* study eight times. Five of the 17 unsupportive reviews cited only evidence that supported their position. Three reviews cited only the supportive trial, implying a difference of interpretation between the reviewers and the authors of that study. Therefore, in total eight reviews (47%) exhibit citation bias for trials of a particular outcome. Three reviews cited just one RCT and two cited all four; most (nine) cited two of the RCTs.

### Supportive reviews

**Fig 4:**
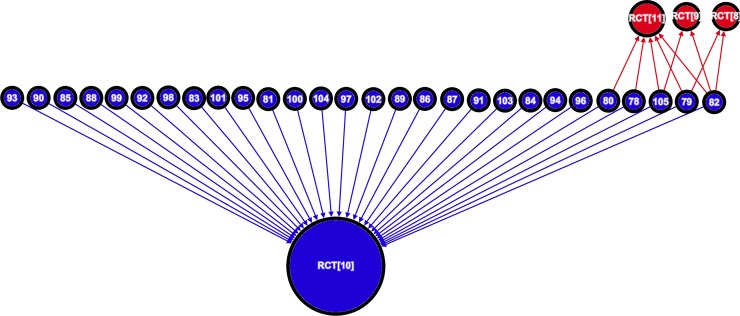
Sub-graph mapping the citations from supportive reviews to RCTs testing cholesterol lowering diets in the secondary prevention of CHD (*n* = 32; *m =* 37). This graph is composed of four RCTs connected to 28 citing reviews by a total of 37 citations. Each vertex represents a paper and the integer label corresponds to a paper in the reference list. The colour of a vertex corresponds to a study’s classification (blue is supportive and red is unsupportive). Vertex size is proportional to in-degree (total number of incoming citations). Each edge corresponds to a specific citation link, the direction of the edge is established by the direction of the arrow head, and the colour of an edge indicates the classification of the cited article.

Compared with the neutral and unsupportive reviews, the 28 supportive reviews (Fig 4) used much less of the available primary data (*D* = 0.33 vs 0.56 in unsupportive reviews and 0.68 in neutral reviews). All 28 cited the single supportive RCT, the *Oslo* study, while the three other RCTs were cited only nine times. In all, 23 of the supportive reviews (82%) cited *only* the *Oslo* study, and only five of these reviews cited *any* of the other RCTs (two cited one other, two cited two others, and just one cited all four RCTs).

Table 1 gives the distribution of citations to RCTs from different classifications of review.

**Table 1:** Contingency table of the distribution of citations from three different classifications of review to four secondary prevention RCTs.

	*Oslo [10]*	*MRC [11]*	*Rose [8]*	*Research Committee [9]*	*Row Totals*
*Neutral*	17	15	7	7	*46*
*Unsupportive*	12	12	6	8	*38*
*Supportive*	28	5	2	2	*37*
*Column Totals*	*57*	*32*	*15*	*17*	*121*

A Pearson’s chi square test was performed to test whether this distribution is significantly related to classification or the product of chance. The validity of a chi square test depends on the conditions that the expected count is greater than 5 for at least 80% of the cells, and that each cell has a count of at least 1: these conditions were both satisfied. The test result establishes that the difference observed in the citation behaviours between reviews is significant (Χ ^2^(6, *N* = 121) = 18.19, *p* = 0.006).

## Discussion

This study examined the propagation of experimental evidence derived from RCTs in reviews of the literature during a period of scientific controversy. This was not a minor academic dispute; this was an issue of considerable public health importance at the time. It documented a major divide between reviews over what the available empirical evidence was, how it should be evaluated, and what the implications were for dietary intervention in the secondary prevention of CHD. Overwhelmingly, reviews that disseminated a supportive evaluation of the results of RCTs in the context of secondary prevention cited only data that supported this position. They did not critique the discrepant data, they simply ignored it, or were ignorant of its existence. Other reviews were overall much more balanced in their citation and this difference in citation behaviour appears to explain divergence of opinion on this matter.

While the *Oslo* trial, a supportive RCT, was by far the most influential, its influence was not uniform amongst different classifications of review. This was the only RCT to arrive at statistically significant findings. A number of studies have reported evidence of a bias in the literature, in that studies with statistically significant results tend to be cited more often than studies that report non-significant or equivocal results [21, 111–114]. However, such a bias for significant outcomes fails to explain the discrepancies between the citing behaviours of different groups.

The degree of utilisation of primary data by reviews appeared to be mediated by a review’s position, with supportive reviews likely to use less of the available RCT evidence than others. Again, this may be explained by a preference for statistically significant findings, but, as discussed, there are problems with this explanation. An alternative hypothesis may be that the shape of the literature itself determines the selection of RCT evidence–where reviewers find evidence from other papers. In this case, if a reviewer believes another paper to have identified all relevant evidence, they may replicate selective citation. This has been described by Greenberg’s information cascade hypothesis–where chains of citations from one paper to another lead researcher’s to only a segment of the available evidence, if those authors simply follow bibliographies [20]. This hypothesis may be supported by Simkin and Roychowdhury’s [115] finding that on average only 20% of the cited literature in bibliographies has been read by the authors. If scientists do tend to gain their information about primary studies by reading other papers rather than the originals, this is particularly worrying in this case due to the amount of bias in the selection of evidence by reviews and the degree of underutilisation. Collectively, the citing reviews studied in this paper have been cited 2,157 times (by August 2017) according to *Web of Science* (supportive reviews 651; neutral 489; unsupportive 1017); however, without further analysis, no statement can be made about what influence these reviews had on the literature.

Thirdly, citation bias was considerably more prevalent in supportive reviews. This seems to be an example of *homophily—*the tendency for a vertex to share an edge with a vertex with which it shares an attribute, and is consistent with Heider’s [116] theory that individuals choose relationships that reinforce existing preferences and beliefs. However, this fails to explain the unsupportive reviews, which we would expect to only cite evidence that supports their perspective. We did not observe this.

Fourthly, few reviews cited all relevant data–with underutilisation common. In the period concerned, conventions governing systematic search strategies were in their infancy. The reviews were mostly of the narrative form, a type that has been criticised for selection bias and the promotion of partial perspectives of the evidence. Indeed, one finding of this paper is that reviews in this field were poor at considering all the available empirical evidence. Bushman and Wells [117] offer experimental evidence of the role of availability bias (a heuristic that describes the tendency of humans to judge the prevalence/probability of an event by the ease in which they are able to recall certain information), which the authors argue influence the propensity for selection bias in narrative reviews compared with systematic reviews and meta-analysis, which impose a rigorous structure of literature searching and study evaluation on the researcher. Importantly, narrative reviews are not the only papers in which citation bias has been documented, Robinson and Goodman [118] documented significant citation bias amongst RCTs addressing the same question and may be explained by the lack of systematic search strategies amongst these papers.

Finally, did the selection of evidence itself explain the divergence of opinion amongst reviews? That is, were these authors only aware of the data they cited? If so, it would seem those underutilising the literature arrive at positions that are based on partial perspectives. Or did pre-existing opinions held by reviewers determine their selection of evidence? That is, were opinions formed first and evidence selected afterwards? If so, this would be a case of confirmation bias. Here, we see the complexities involved in attempting to determine which direction the arrow of causality points–is evidence formative in scientific opinion? Or do pre-existing opinions dictate the evidence we choose to examine?

The major findings offer an alternative to the prevailing notion of what a scientific controversy *is*, i.e. that scientific disagreement stems from different theoretical positions, where researchers looking at the *same* evidence can justifiably come to different conclusions [119]. Disagreement in this instance appears to stem from the selection of *different* sources of evidence, rather than the possession of different theoretical perspectives. Trinquart *et al*. [27] came to similar conclusions when examining the controversy about the suggested benefits of restricting dietary salt intake. They observed “two almost distinct and disparate lines of scholarship, one supporting and one contradicting the hypothesis” (p.57)–lines that relied on different sources of evidence and which differed in their citing behaviour.

### Implications

Scientists, at present, are concerned at a wide range of factors that they understand to be distorting science. These include publication bias [120, 121], problems with replication [122], and citation biases [20, 21, 27, 111–114]. Their combined effect has undermined faith in the idea that our sciences are self-correcting, and increasingly commentators fear that our system of knowledge production is broken. De Solla Price [123] and later Ravetz [124] raised concerns that as science grew it may experience ‘senility’–with the evidence base and citing literature growing too large for effective quality control, but it is a view that is gaining increasing attention [125].

This study focused on citation bias, a structural bias that leads to distortions of authority, whereby some positions are held up by the inflated authority of supporting primary studies and where scientists do not interact with, and in some cases may be unaware of, rival perspectives and unsupportive data [20]. This can lead to polarisation, where different scientists draw from different sources of evidence and arrive at opposing conclusions to similar questions [27]. Unlike these two studies, which examined recent citation practices, the present study focused on a historic analysis of divergent citation practices amongst papers drawing different conclusions as to what the available empirical evidence supports. Citation bias and research underutilisation were exactly documented, as were the different citation behaviours of different classifications of review.

This case study was chosen because it represents a unique example where the relevant RCTs were published in quick succession (1965–1968), and these remained the only RCTs addressing this particular question prior to a major consensus building conference in 1984. This provided a 17-year citation window, from the time all results of the RCTs were available to researchers to the beginning of this conference, where the evidence-base, as derived from RCTs, remained constant (i.e. no additional secondary prevention RCTs were published that would have impacted the evidence-base and, possibly, citation behaviours). Further, as these RCTs were the first to test this particular treatment, the relevant evidence-base and citation behaviour could not have been influenced by prior studies, at-least not evidence derived from RCTs, which may have distorted the results. This provided a unique opportunity to study how scientists utilise a small evidence-base over a long and undisturbed period. One surprising findings is that, even with a small evidence-base, scientists did not generally cite these studies in a representative manner nor did their citation behaviours improve as this period unfolded, and this challenges our understanding that it may be the size of the literature responsible for this phenomenon.

Therefore, the major findings of this study demonstrate that citation bias is not a new phenomenon–challenging narratives that suggest our science has become increasingly less reliable in recent years because science has grown too large–and that scientific disagreement may revolve around something more simple than divergent theoretical positions–scientists may just be looking at different sources of data.

The results of this study should not be taken as making claims about the current state of knowledge regarding the efficacy of dietary intervention in secondary prevention. Nor should it be used to fuel the current controversy over scientific advice concerning dietary fat consumption. Indeed, the selection of a historical cases study was, in part, chosen to avoid this paper being mistaken for attempting to weigh in on what particular treatment is suitable in secondary prevention. Since the 1980s, much work has been conducted in this area and the results of this study do not impact the scientific case. Rather, the aim was to provide a deeper understanding of the ways in which empirical data is used by scientists, how it may fuel scientific disagreement, and to introduce a method capable of detecting this particularly efficiently. Its focus was on citation practices and evidence dissemination, not the validity of current scientific theories.

## Supporting information

S1 Table Study characteristics of four RCTs examining dietary fat restriction/modification in the secondary prevention of CHD. A comparison of the Oslo Diet–Heart Study, Rose Corn Study, Research Committee Low-fat Study, and Medical Research Council’s MRC Soya-bean Oil Trial.
(DOCX)

S2 Table Intervention and diet characteristics of four RCTs examining dietary fat restriction/modification in the secondary prevention of CHD. A comparison of the Oslo Diet–Heart Study, Rose Corn Study, Research Committee Low-fat Study, and Medical Research Council’s MRC Soya-bean Oil Trial.
(DOCX)

S3 Table A comparison of the incidence of myocardial infarction, CHD mortality (myocardial infarction + sudden death), combined fatal + non-fatal CHD events, all-cause mortality, and changes in total serum cholesterol in four secondary prevention RCTs. A comparison of the Oslo Diet–Heart Study, Rose Corn Study, Research Committee Low-fat Study, and Medical Research Council’s MRC Soya-bean Oil Trial.
(DOCX)

S4 Table A risk-ratio analysis of the results of four secondary prevention RCTs. A comparison of the Oslo Diet–Heart Study, Rose Corn Study, Research Committee Low-fat Study, and Medical Research Council’s MRC Soya-bean Oil Trial.
(DOCX)

S5 Table A quotation analysis of four RCTs and 62 citing reviews. (DOCX)

S6 Table Attribute data on all studies (vertices) included in this study. (DOCX)

S7 Table Edge data on all citations between reviews and RCTs included in this study. (DOCX)
