# Supplementary material for: A network analysis of the propagation of evidence regarding the effectiveness of fat-controlled diets in the secondary prevention of coronary heart disease (CHD): Selective citation in reviews
Source: PLoS One. 2018 May 24;13(5):e0197716. doi: 10.1371/journal.pone.0197716 (PMC5968408; doi:10.1371/journal.pone.0197716)
Supplement: S1 Table — A comparison of the Oslo Diet–Heart Study, Rose Corn Study, Research Committee Low-fat Study, and Medical Research Council’s MRC Soya-bean Oil Trial. (DOCX) [file pone.0197716.s001.docx]

**Supplementary Material**

**Introductory note:**

S1–4 Tables contain only the information that was published about the four secondary prevention RCTs analysed in this study. The sample characteristics, study characteristics, and results were extracted from these original publications and no effort was made to recover lost data or to include data that others may have recovered. Accordingly, the results of the risk ratio (RR) analysis in S4 should not be taken as indicative of the current state of knowledge about either these trials or the efficacy of dietary treatment in secondary prevention. Rather, these results exhibit the information that would have been available to reviewers during the citation window examined (1969–1984). The information for Tables S1–3 were extracted from the original trial publications. For the analysis (S4 Table), the raw data on mortality and morbidity (S3 Table) was analysed via a RR analysis in Microsoft Excel. S5 Table contains quotation data extracted from the original publications of every paper examined in this study, the classification of each paper that this quotation substantiated, and a comment box containing additional explanatory notes. S6 Table provides the attribute information for all papers (vertices), and S7 Table provides all data on edges (citations) between papers in this set, and these are necessary to reconstruct the graphs analysed in this paper.

**S1 Table. Study characteristics of four RCTs examining dietary fat restriction/modification in the secondary prevention of CHD.** A comparison of the Oslo Diet – Heart Study, Rose Corn Study, Research Committee Low-fat Study, and Medical Research Council’s MRC Soya-bean Oil Trial.

| Trial | Type of Study | Randomised | Blinding | Total Duration of Study, years | Mean years in study intervention/  control | No. of individuals in intervention/ control | Mean age, years, intervention/ control | Outcome measures |
| --- | --- | --- | --- | --- | --- | --- | --- | --- |
| Rose Corn Trial [8] | DI,U,MF, SP | Yes | SBI | 2 | 1.5/1.7 | 28/26 | 53/59 | TC, MI, CHD, All-cause mortality |
| Rose Olive Trial [8] | DI,U,MF, SP | Yes | SBI | 2 | 1.5.1.7 | 26/26 | 55/59 | TC, MI, CHD, All-cause mortality |
| Research Committee Low-Fat [9] | DI,U,RF,SP | Yes | SBI | 4 | 3.04/3.05 | 123/129 | <65/<65 | TC, MI, CHD, All-cause mortality |
| MRC Soybean Oil [11] | DI,U,MF,SP | Yes | SBI | 4 | 3.8/3.7 | 199/194 | <60/<60 | TC, MI, CHD, All-cause mortality |
| Oslo-Diet Heart [10] | DI,U,MF,SP | Yes | SBI | 5 | 4.3/4.3 | 206/206 | 56/56 | TC, MI, CHD, All-cause mortality |

DI – Dietary intervention; U – Unifactorial; MF – Modified fat; RF – Restricted fat; SP – Secondary prevention; SBI – Single blinding of investigators only; TC – Total cholesterol; MI – Myocardial infarction (both fatal and non-fatal); CHD – Coronary heart disease (including MI, sudden cardiac death, and angina).
