# Supplementary material for: A network analysis of the propagation of evidence regarding the effectiveness of fat-controlled diets in the secondary prevention of coronary heart disease (CHD): Selective citation in reviews
Source: PLoS One. 2018 May 24;13(5):e0197716. doi: 10.1371/journal.pone.0197716 (PMC5968408; doi:10.1371/journal.pone.0197716)
Supplement: S2 Table — A comparison of the Oslo Diet–Heart Study, Rose Corn Study, Research Committee Low-fat Study, and Medical Research Council’s MRC Soya-bean Oil Trial. (DOCX) [file pone.0197716.s002.docx]

**S2 Table. Intervention and diet characteristics of four RCTs examining dietary fat restriction/modification in the secondary prevention of CHD.** A comparison of the Oslo Diet–Heart Study, Rose Corn Study, Research Committee Low-fat Study, and Medical Research Council’s MRC Soya-bean Oil Trial.

| Trial | Dietary treatment | Intervention | Method of issuing intervention advice and assessment. | Fat, g (% of calories) | SFA/MUFA/PUFA composition % of calories^a^ |
| --- | --- | --- | --- | --- | --- |
| Rose Corn Trial [8] | MF | Intervention: Corn-oil supplementation (80g/day) and advice to restrict SFA.  Control: No dietary advice given | Dietary advice from physicians to free-living population and a monthly follow-up clinic. Free oil and advice given to all participants. | 116 (50%)/  70 (30–32%) | Intervention: 14/ 14.9/ 21.85/  Control: 16–18/ 7/ 7 |
| Rose Olive Trial  [8] | MF | Intervention: Olive-oil supplementation (80g/day) and advice to restrict SFA  Control: No dietary advice given | Dietary advice from physicians to free-living population and a monthly follow-up clinic. Free oil and advice given to all participants. | 105 (46%)/  70 (30–32%) | Intervention: 11.7/ 26/ 8.7  Control: 16–18/ 7/ 7 |
| Research Committee Low-Fat [9] | RF | Intervention: Patients advised to eat no more than 40g/per day of fat. The daily allowance included 14 g (1/2 oz) butter, 84 g (3 oz) of meat, 1 egg, 56 g (2 oz) cottage cheese, and skimmed milk.  Control: No dietary advice given | Dieticians issued advice directly to free-living patients. Patient and wife interviewed by the doctor + dietitian at first outpatient visit, 2 weeks from leaving hospital. Patient attended every 2 weeks for 3 months, every 3 months for 2 years, and every 6 months thereafter. | 44 (20%)/  112 (42%) | Intervention: 9/ 8/ 5  Control: 16–18/ 15/ 7 |
| MRC Soybean Oil [11] | MF | Intervention: Soya-bean oil supplementation (85g/day) and advice to remove dietary SFA and other dietary fat to under 35 g/day  Control: No advice given. | Free living Patients + wives interviewed by doctor + dietitian at his first outpatient visit 2 weeks from start of trial, and the instructions were given. Follow-ups were made every 2 weeks for the first 2 months, then at 3 months and every 3 months thereafter. | 120 (46%)/  115 (45%) | Intervention: 11.3/ 14.3/ 20.4  Control: 26.4/ 12.2/ 4.4 |
| Oslo-Diet Heart [10] | MF | Intervention: Soya-bean oil supplementation (72g/day from weekly 0.5 L allocations). Vitamin tablet supplementation. Cod liver oil + sardines supplementation. Advice to reduce dairy and meat products, increase fish and vegetable consumption.  Control: No advice given. | Adherence to the diet was controlled by close personal contact, the physician being assisted by a full time, experienced dietician who worked in the homes of the patients. The degree of adherence was quantified by means of a detailed questionnaire used six times during the period of observation. 25% of patients received free soya-bean oil. | 104 (39%)/  NA | Intervention: 6.5/ 10.1/ 20.7 Control: 16–18/ 15/ 7 |

MF – Modified fat; RF – Restricted fat; SFA – saturated fatty acids; PUFA – polyunsaturated fatty acids; MUFA – monounsaturated fatty acids.

^a^Figures taken from Schwingshackl and Hoffmann [18] (pp. 5–6)
