# Supplementary material for: A network analysis of the propagation of evidence regarding the effectiveness of fat-controlled diets in the secondary prevention of coronary heart disease (CHD): Selective citation in reviews
Source: PLoS One. 2018 May 24;13(5):e0197716. doi: 10.1371/journal.pone.0197716 (PMC5968408; doi:10.1371/journal.pone.0197716)
Supplement: S3 Table — A comparison of the Oslo Diet–Heart Study, Rose Corn Study, Research Committee Low-fat Study, and Medical Research Council’s MRC Soya-bean Oil Trial. (DOCX) [file pone.0197716.s003.docx]

**S3 Table. A comparison of the incidence of myocardial infarction, CHD mortality (myocardial infarction + sudden death), combined fatal + non-fatal CHD, all-cause mortality, and changes in total serum cholesterol in the four secondary prevention RCTs.** A comparison of the Oslo Diet–Heart Study, Rose Corn Study, Research Committee Low-fat Study, and Medical Research Council’s MRC Soya-bean Oil Trial.

| Trial | No. patients experiencing a fatal + non-fatal MI  [Interv/Control] | No. patients with fatal CHD (MI & sudden death)  [Interv/Control] | No. patients with fatal/non-fatal CHD (MI/sudden death/ angina pectoris)  [Interv/Control] | No. all-cause mortality  [Interv/Control] | Mean initial TC, mmol/L  [Interv/Control] | Mean TC level over trial period, mmol/L  [Interv/Control] | Mean mmol/L TC change, %  [Interv/Control] |
| --- | --- | --- | --- | --- | --- | --- | --- |
| Rose Corn Trial [8] | 9/5 | 5/1 | 15/11 | 5/1 | 6.8/6.54 | 6.12/6.5 | –10.0/–0.61 |
| Rose Olive Trial  [8] | 7/5 | 3/1 | 11/11 | 3/1 | 6.78/6.54 | 6.89/6.5 | +1.6/–0.61 |
| Research Committee Low-Fat [9] | 31/34 | 17/20 | 46/48 | 20/24 | 6.72/6.88 | 5.67/ 6.25 | –15.6/–9.2 |
| MRC Soybean Oil [11] | 45/51 | 25/25 | 62/74 | 28/31 | 7.03/7.06 | 5.86/6.73 | –16.6/–4.8 |
| Oslo-Diet Heart (5 years) [10] | 34/54 | 37/50 | 64/90 | 41/55 | 7.65/7.65 | 6.31/7.37 | –17.5/–3.7 |
| Total (All) | 126/144 | 87/96 | 198/223 | 97/111 |  | | |
| Total (excluding Rose olive due effect on TC) | 119/144 | 84/96 | 188/223 | 94/111 |  |  |  |
