# Supplementary material for: A network analysis of the propagation of evidence regarding the effectiveness of fat-controlled diets in the secondary prevention of coronary heart disease (CHD): Selective citation in reviews
Source: PLoS One. 2018 May 24;13(5):e0197716. doi: 10.1371/journal.pone.0197716 (PMC5968408; doi:10.1371/journal.pone.0197716)
Supplement: S4 Table — A comparison of the Oslo Diet–Heart Study, Rose Corn Study, Research Committee Low-fat Study, and Medical Research Council’s MRC Soya-bean Oil Trial. (DOCX) [file pone.0197716.s004.docx]

**S4 Table. A risk ratio (RR) analysis of the results of four secondary prevention RCTs.** A comparison of the Oslo Diet–Heart Study, Rose Corn Study, Research Committee Low-fat Study, and Medical Research Council’s MRC Soya-bean Oil Trial.

| Trial | RR (95 % confidence interval)  <1 favours intervention >1 favours control | | | |
| --- | --- | --- | --- | --- |
|  | All MI | Fatal CHD (MI & sudden death) | Combined fatal/non-fatal cardiovascular events. | All-cause morality |
| Rose Corn Trial [8] | 1.67  (0.64 – 4.34) | 4.64  (0.58 – 37.1) | 1.27  (0.72 – 2.23) | 4.64  (0.58 – 37.1) |
| Rose Olive Trial [8] | 1.4  (0.51 – 3.85) | 3  (0.3 – 27) | 1  (0.53 – 1.90) | 3  (0.33 – 27) |
| Research Committee Low-Fat [9] | 0.96  (0.63 – 1.45) | 0.89  (0.49 – 1.62) | 1.0  (0.73 – 1.38) | 0.87  (0.51 – 1.5) |
| MRC Soybean Oil [11] | 0.86  (0.61 – 1.22) | 0.97  (0.58 – 1.64) | 0.84  (0.64 – 1.11) | 0.88  (0.55 – 1.41) |
| Oslo-Diet Heart (5 years)  [10] | **0.63**  **(0.43 – 0.92)** | 0.74  (0.51 – 1.08) | **0.71**  **(0.55 – 0.92)** | 0.75  (0.52 – 1.1) |
| Total (All studies) | 0.83  (0.68 – 1.03) | 0.86  (0.66 – 1.13) | **0.85**  **(0.73 – 0.99)** | 0.83  (0.65 – 1.06) |
| Total (Excluding Rose Olive) | 0.82  (0.67 – 1.02) | 0.87  (0.67 – 1.14) | **0.84**  **(0.72 – 0.98)** | 0.84  (0.66 – 1.1) |

The above table (S4 Table) contains results of a RR analysis performed in Microsoft Excel of the raw mortality and morbidity data contained in the above table (S3 Table). RR was established by

$$RR=\frac{a/(a+b)}{c/(c+d)}$$

where *a* is the number in the exposed group with a disease, *b* the number in the exposed group without a disease, *c* the number in the unexposed group with a disease, and *d* the number without a disease in the unexposed group.

To establish the standard error of RR, we convert to a natural log (ln) scale

$$SE\left\{ ln\left( RR \right) \right\}= \sqrt{\frac{1}{a}+\frac{1}{c}-\frac{1}{a+b}-\frac{1}{c+d}}$$

and we establish a 95% confidence interval by taking the antilog (exp) of the lower and upper limits by

$$95\% CI=\exp(ln\left( RR \right)-1.96 x SE\left\{ ln\left( RR \right) \right\} to\exp(ln\left( RR \right)+1.96 x SE\{ln\left( RR \right)\}$$
