# Supplementary material for: A network analysis of the propagation of evidence regarding the effectiveness of fat-controlled diets in the secondary prevention of coronary heart disease (CHD): Selective citation in reviews
Source: PLoS One. 2018 May 24;13(5):e0197716. doi: 10.1371/journal.pone.0197716 (PMC5968408; doi:10.1371/journal.pone.0197716)
Supplement: S5 Table — (DOCX) [file pone.0197716.s005.docx]

**S5 Table: A quotation analysis of four RCTs and 62 citing reviews.**

| Vertex ID + Reference | Author | Quote | Classification | Comment |
| --- | --- | --- | --- | --- |
| **RCTS** | | | | |
| RCT1  [9] | Research Committee Low-fat | A low-fat diet has no place in the treatment of myocardial infarction (p. 504) | Unsupportive | Clearly unsupportive and interpretation accurately reflects study results. |
| RCT2  [8] | Rose, GA; Thomson, WB; Williams RT | It is concluded that under the circumstances of this trial corn oil cannot be recommended in the treatment of ischaemic heart disease (p. 1533) | Unsupportive | Clearly unsupportive and interpretation accurately reflects study results |
| RCT3  [10] | Leren, P | The reduction of the serum cholesterol level associated with a reduced CHD relapse rate strongly suggests a cause and effect relationship (p. 79). | Supportive | Supportive trial – author fairly summarises results and suggests these are evidence in support of causal relationship. |
| RCT4  [11] | Medical Research Council | …the results of this trial alone lend little support to this suggestion or to the suggestion that a diet of the kind used should be recommended in the treatment of patients who have suffered a myocardial infarction (p. 699) … Taken together with the results of the Oslo trial (Leren, 1966) there is no indication that this type of diet affects mortality. The combined evidence, however, suggests that a proportion of non-fatal reinfarctions might be prevented, though more evidence is required to confirm this effect, and to make a useful estimate of its size. | Unsupportive | Unsupportive based on the findings and conclusion of only this study. Accurately reflects study findings. The directors provide a small comparison with Leren’s (1966) study and suggest that the combined results might suggest a benefit in the reduction on non-fatal CHD events. |
| **Unsupportive Reviews** | | | | |
| R3  [74] | May, GS; Eberlein, K; Furberg, CD; Passamani, ER; DeMets DLPassamani, and David L. DeMets | The overall impression after considering each of the nine trials [2 of which were dietary trials, the rest were drug trials] (Leren, 1966; Leren 1970; Medical Research Council 1968) ... is that lipid- lowering does not prolong life in the post-MI population studied. (p. 339) | Unsupportive | No evidence of citation bias – cites 2/4 available dietary secondary prevention RCTs.  Mixes drug and diet intervention studies together in evaluation |
| R7  [73] | Oliver, MF | Thus, dietary trials and drug trials have not shown a significant reduction of non-fatal or fatal myocardial infarction with any consistency…The result of two of the major secondary prevention trials (Medical Research Council, 1968; Leren 1966) of diets with polyunsaturated fats are in conflict and no large formal trial of a diet low in saturated fats has ever been done. My view is that reduction of raised serum cholesterol once IHD has presented is of little or no benefit. The recent recommendation of the International Society and Federation of Cardiology (1981) for "secondary prevention in survivors of myocardial infarction" that serum cholesterol should be reduced to the region of 4–5 mmol/L (175 mg/dL) is not supported by the available evidence, is without proof of benefit, and is extremely difficult to achieve so far as serum lipids are concerned. (p. 1094) | Unsupportive | Cites the two largest studies, which give a sense of the conflicting nature of trial findings. No evidence of citation bias and cites 2/4 RCTs. |
| R6  [69] | McMichael, J | The best-conducted dietary trials under the auspices of the MRC's statistical control have given convincingly negative results. In survivors who have had coronary manifestations, and are thus at special risk, low-fat (Research Committee, 1965) and soy-bean oil diets (Medical Research Council, 1968), which can lower the blood cholesterol concentration, have been entirely ineffective in slowing the progress of the disease towards recurrences or death (p. 173) | Unsupportive | Citation bias favouring unsupportive trials and cites 2/4 available RCTs. |
| R53  [67] | Mann, GV | Clinical trials of prevention by diet, both primary (Dayton and Pearce1964) and secondary (Leren, 1966; Miettinen et al. 1972), have shown that diet has than a trivial effect on cholesteremia and has no real effect on morbidity and mortality (p. 569) | Unsupportive | Citation bias favouring supportive trials – cites 1/4 available RCTs.  The author cites only supportive trials in this unsupportive statement. The assessment is erroneous, Leren (1966) found a significant reduction in fatal MI and a significant reduction in CVD events. Miettinen et al. (1972), otherwise known as the *Finnish Mental Hospital Trial*, was not an RCT – it was a “cross-over” trial in two mental hospitals. Further, it was not a standard cross-over trial: the patient groups were not the same in the diet arm and the control arm. No blinding mentioned (important because the investigators re-coded deaths). Finally, it was a combined primary and secondary prevention trial, and in the paper cited, it is not possible to extract findings regarding secondary prevention specifically.  The paper cited for Dayton and Pearce (1964) is a referencing error. The paper in question is a review published in 1969 – not the original publication of the trial results of their *LA Veterans Study*. |
| R5  [66] | Mann, GV | No diet therapy has been shown effective for the prevention or treatment of coronary heart disease (Leren et al. 1966; Dayton and Pearce 1964; Miettinen et al. 1972). (p. 646) | Unsupportive | Citation bias favouring supportive trials and cites 1/4 available secondary prevention RCTs.  Erroneous interpretation of studies cited. Leren (1966) found significant beneficial effects of dietary intervention. Miettinen et al. (1972) found supportive evidence. Dayton et al. (1968) also found supportive evidence that dietary intervention might CVD events.  Mann replicates the original citation error [67] with the Dayton and Pearce paper (1964). |
| R48  [70] | McMichael, J | An immense effort has been devoted to the reduction of cholesterol levels in the blood by diet (Research Committee, 1965; Medical Research Council, 1968) and by drugs, and it must now be concluded that these efforts have had no detectable influence on the course or development of coronary heart disease. (p. 409). | Unsupportive | Bias favouring unsupportive trials and cites 2/4 available secondary prevention RCTs. |
| R47  [77] | Oliver, M F | The results of dietary trials after myocardial infarction are also conflicting with two negative small British studies (Research Committee, 1965; Rose et al. 1965) and a positive open, non-blind Norwegian trial (Leren, 1966), where a low saturated fat and high polyunsaturated fat diet was reported to reduce the incidence of recurrent myocardial infarction and coronary deaths in men under 60 with initially high concentrations of serum cholesterol (7.7 mmol/L; 296 mg/100 mL): a 14% difference between the diet and control groups was achieved over the five years of this study. Without clearer, more convincing evidence of benefit **we are not justified** in imposing on all patients dietary or drug regimens which many find unpleasant. (p. 1641) | Unsupportive | No evidence of citation bias, cites 3/4 secondary prevention RCTs, and provides a balanced discussion. |
| R40  [62] | Lees, RS; Wilson D E | It may be valid to exert maximal therapeutic efforts with young patients with severe hyperlipoproteinema uncomplicated by clinical coronary disease. It is probably unwarranted to force such efforts, which entail major changes in diet and lifestyle…upon middle-aged men with severe establish coronary disease. Available evidence suggests that little or no benefit is to be gained in such cases (Leren, 1966; Turpeinen et al. 1968; Medical Research Council, 1968; Dayton et al. 1969). (p. 190) | Unsupportive | No evidence of citation bias, cited 2/4 available secondary prevention RCTs.  Cites Turpeinen et al. 1968, which refers to the *Finnish Mental Hospital Trial*. This is an earlier report of the same trial Miettinen et al. (1972) reported. |
| R4  [65] | Borhani, NO | …among secondary prevention trials are those conducted in Great Britain (Rose et al. 1965, Medical Research Council, 1968) Norway (Leren, 1966). The results obtained in these trials confirm what had been demonstrated convincingly in the past, namely, that plasma lipid levels can be intentionally altered. However, the data were either negative or at best equivocal in demonstrating the potential benefits of plasma lipid-lowering measures in prevention of morbid events or mortality, or both. (p. 253) | Unsupportive | No evidence of citation bias and cites 3/4 available secondary prevention RCTs. |
| R38  [64] | Ahrens, EH | In most of these studies (Leren, 1966; Rose et al*.* 1965; Medical Research Council 1968; Christakis 1966; Bierenbaum et al. 1973; Miettinen et al. 1972; Frantz et al. 1975) claims were made for benefit in terms of new events of coronary heart disease; however, serious objections have been raised to these claims in every case. (p. 89)…In summary, then, the results obtained in these intervention trials (primary or secondary, diet or drugs) are equivocal at best; they lead us to no clear cut conclusion that the effort was worth the cost. (p. 89) | Unsupportive | No evidence of citation bias and cites 3/4 available secondary prevention RCTs.  Mixes secondary and primary prevention trials together in evaluation and RCTs and non-RCTs. Chistakis (1966) was a primary prevention non-RCT, Frantz et al. (1975) was a combined primary and secondary prevention trials, and this publication does not report the results for those with pre-existing CHD separately. This is another example of partial publication – Ramsden et al. (2016) recently recovered the results. Bierenbaum et al. (1973) is a non-RCT in secondary prevention – assigning a control group post hoc. |
| R34  [61] | Dayton, S; Pearce ML | In summary, critical weighing of all the secondary prevention trials is far from convincing as to the clinical usefulness of diet in those who survive myocardial infarction…Three of these potentially less biased trials found the cholesterol-lowering diet clinically ineffective (Rose et al. 1965; Research Committee 1965) or nearly so (Medical Research Council 1968). The fourth -the trial conducted by Leren (1966) in Oslo - involved blindfold evaluation of myocardial infarction but not of angina pectoris…We concur with the conclusion of the most recent of these that there is “little support . . . to ‘the suggestion that a diet of the kind used [high in unsaturated fat] should be recommended in the treatment of patients who have suffered a myocardial infarction…The combined evidence, however, suggests that a proportion of nonfatal reinfarctions might be prevented though more evidence is required to confirm this effect . . .” (Medical Research Council 1968) (p. 760) | Unsupportive | No evidence of citation bias and cites all available secondary prevention RCTs. |
| R29  [68] | Mann, JI | Nevertheless, here too I consider that Professor McMichael may perhaps have misled his readers: "The best conducted dietary trials under the auspices of the MRC's statistical control have given convincingly negative results." The trials (Research Committee 1965; Medical Research Council 1968) to which he refers are "secondary prevention" studies in which prevention is attempted in individuals who have already experienced an ischaemic episode. The results, while disappointing, are perhaps not surprising. In such subjects atherosclerosis is already well established and the subjects are no longer young, so it might be a case of trying to shut the stable door at rather too late a stage (p. 733) | Unsupportive | Citation bias favouring unsupportive trials – cites 2/4 RCTs and only unsupportive trails.  Author agrees with McMichael [70] that dietary intervention may be ineffective in those with established CHD. |
| R26  [72] | Inkeles, S; Eisenberg, D | Clinical Trials have involved populations with no previous evidence of CHD (Dayton et al, 1969; Turpeinen et al. 1968) as well as those with diagnosed CHD (Leren, 1970; Bierenbaum et al. 1973)…Although some decrease in incidence and mortality rates of CHD were noted, the results of these studies have failed in general to demonstrate a clearly beneficial effect (p.117)…Mann, in a recent critique of these diet trials concluded that: “No diet therapy has been shown effective for the prevention or treatment of coronary heart disease”. By making such a sweeping statement, however, Mann erroneously implies that dietary intervention in general is ineffective in either the prevention or treatment of CHD…the conclusion can only be applied to diets of this particular make-up – i.e. low in saturated fat, while still relatively high in total fat content (p. 117) | Unsupportive | Citation bias favouring supportive evidence – cites 1/4 available secondary prevention RCTs.  Also cites non-RCTs - Bierenbaum et al (1973), Turpeinen et al. (1968). Also cites Dayton et al. (1969) mixed secondary and primary prevention RCT, which did not report full results regarding only secondary prevention – therefore, relevant only to primary prevention. |
| R22  [63] | Carnie, J A | …trials of the second type [Secondary Prevention] have been less encouraging and do not indicate that a low fat diet supplemented with unsaturated fats, or a diet low in saturated fats have a significant effect on the course of IHD. (Research Committee 196; Rose et al. 1965) (p. 235) | Unsupportive | Citation bias favouring unsupportive trials (2/3). Does not cite the supportive trial of Leren (1966). |
| R2  [76] | Coates, ME | Secondary intervention trials have shown little evidence of benefit from a change in diet after a coronary event has been experienced (Leren, 1966; Medical Research Council 1968; Bierenbaum et al. 1973)…Most intervention trials have been started on middle-aged subjects in whom arteriosclerotic lesions have probably begun to form. Regression at this stage would be unlikely to be complete, especially in those who have already suffered a cardiac event, whereas the prognosis is likely to be more favourable in younger subject (p. 551–552) | Unsupportive | Author suggests that dietary intervention is unlikely to be beneficial to those with CHD. Cites 2/4 available secondary prevention RCTs. Also cites, Bierenbaum et al. (1973) inadequately controlled trial. |
| R13  [71] | Walker, ARP | Although secondary prevention trials have been undertaken [cites two dietary (Medical Research Council 1968; Research Committee 1965) and one drug trial] as J. I. Mann (1979) has commented: 'The results, while disappointing, are perhaps not surprising. In such subjects atherosclerosis is already well established and the subjects are no longer young, so it might be a case of trying to shut the stable door at rather too late a stage.' (p. 11) | Unsupportive | Author concurs with JI Mann that dietary intervention in those with CHD is unlikely to be of benefit.  Citation bias favouring unsupportive trials (2/3) – does not cite Leren (1966) |
| R1  [75] | Buckwald, H; Fitch L; Moore, RB | All completed randomized clinical trials [of dietary studies examined – Rose et al. 1965; Research Committee 1965; Leren 1966; Medical Research Council, 1968)] of lipid intervention for atherosclerotic cardiovascular disease have shown no convincing evidence for disease retardation, arrest, or reversal associated with plasma cholesterol reduction, although in no trial has cholesterol reduction been marked and in many it has been miniscule (p. 279) | Unsupportive | Full utilisation of available secondary prevention RCTs. |
| **Neutral Reviews** | | | | |
| R9  [54] | Kritchevsky, D | A secondary prevention trial in Oslo (Leren, 1966) showed that men placed on a diet high in unsaturated fat had fewer myocardial infarctions, but a similar trial in England was without effect (Medical Research Council 1968). (p. 617) | Neutral | Neutral – highlights conflicting nature of trial results. No evidence of citation bias. Research utilised 2/4 RCTs |
| R8  [59] | Goldman, GL; Pichard, AD | The effect of diets, high in polyunsaturated fats and low in saturated fats and cholesterol, on the mortality from coronary disease has been evaluated, but a beneficial influence has not been firmly established. In the Oslo-Diet Heart Study (Leren 1966; Leren 1970), there was a 25% reduction in mortality with 5 years of diet intervention. This was not statistically significant...There was, however, a statistically significant reduction in fatal myocardial infarctions. In the Medical Research Council Trial (1968) diet intervention failed to cause a significant mortality over 5 years of follow up (p. 541) | Neutral | Neutral – highlights conflicting nature of trial results. No evidence of citation bias. Research utilised 2/4 RCTs. |
| R52  [46] | Walker, ARP | Investigations on patients who have had myocardial infarction. The British studies on low fat intake (Research Committee, 1965), alone, or supplemented with corn oil (Rose et al. 1965), or soy-bean oil (Medical Research Council, 1968), all yielded negative results, i.e. there was not a significant difference in re-infarction rate between the experimental and control groups. The USA studies of Morrison (1960) and of Bierenbaum et al. (1967) based on low fat and low cholesterol regimens, revealed an advantage to the experimental groups by virtue of a lower frequency of re-infarctions. This also applied to the investigation of Leren, perhaps the most widely quoted. (p. 771) | Neutral | Neutral – highlights conflicting nature of trial results. No evidence of citation bias. Research utilised 4/4 RCTs. Also, discusses to non-RCTs (Morrison 1960; Bierenbaum 1967). No clear judgement is made about whether the trial evidences supports intervention. |
| R51  [52] | Howard, A N | Two trials which have examined the effect of a prudent diet (high polyunsaturated, low saturated fat and low cholesterol) on the morbidity and mortality of patients with coronary heart disease have given equivocal results. In a trial in Oslo, Leren (1966) found that in 412 subjects lowering serum cholesterol by 14% did not affect the incidence of sudden deaths. However, the number of coronary events were significantly reduced. Likewise in a trial with 373 patients, in which soya bean oil was employed, the difference in serum cholesterol between experimental subjects and controls was 16% and no difference in death rate was established (Medical Research Council, 1968). (p.112). The general conclusion from all these trials is that further work in several thousands of subjects over many years would be necessary to provide a conclusive answer as to whether lowering serum lipids by diet can affect mortality from coronary heart disease. (p. 113) | Neutral | Neutral – highlights conflicting nature of trial results. No evidence of citation bias. Research utilised – 2/4 RCTs. Stresses need for further research. |
| R50  [58] | Hulley, SB; Lo, B | Not yet proven is whether dietary lowering of serum cholesterol levels will help prevent CHD. Of the randomized single-factor trials, some have shown no benefit (Medical Research Council, 1968; Dayton et al. 1968) while others have revealed reductions in atherosclerotic events but not in overall mortality (Leren 1970; Miettinen et al. 1972) (p. 667) | Neutral | No evidence of citation bias – utilises 2/4 available secondary RCTs. Authors’ mix together primary and secondary prevention trials (include Dayton et al. 1968 – relevant to primary prevention) and include the non-RCT Miettinen et al. (1972) study. |
| R41  [55] | Yeshurun, D; Gotto AM | The most frequently given reason for treating hyperlipidemia is the expectation of decreasing the risk of arteriosclerotic cardiovascular events. Numerous studies have dealt with this problem, often with conflicting results. [cites Leren, 1966, but also three drug trials and Christakis et al. (1966) primary prevention dietary trial] (p. 381) | Neutral | Highlights conflicting results with no clear evaluative statement. Citation bias for supportive trials and utilises only 1 of 4 available RCTs. |
| R33  [44] | Cornfield, J; Mitchell, S | It seems clear that despite a very considerable scientific effort and some tantalizingly suggestive results, we have no clear-cut, generally accepted answer to the question of whether cholesterol lowering measures can affect coronary disease (p. 388).  So we end where we started. There are good grounds for believing that certain potentially modifiable risk factors may have an important influence on the amount of coronary disease. The problem is complicated, however. It does not appear as if, despite considerable effort, we have been successful in surmounting difficulties and actually demonstrating the possibility of reduction or of estimating its magnitude. We shall keep trying, but will have to do better (p. 391). | Neutral | This statement comes after a comparative analysis of trials and RCTs. In that analysis, the authors’ cite all four secondary prevention RCTs. Classified as neutral due stress on further research. |
| R20  [45] | Brown, DF | Support for the concept that restriction of dietary fat is of clinical importance is claimed in reports of long-term therapeutic trials with such diets. The studies of Leren (1967), Turpeinen et al. (1968), and Dayton et al. (1968)…suggest that the use of these diets has resulted in definite lipid reduction. They have not shown conclusive evidence of clinical benefit. Other studies of a similar nature have not shown evidence of therapeutic benefit despite “chemical improvement.” (Rose et al 1965; Research Committee 1965; Medical Research Council, 1968) (p. 701). | Neutral | Author stresses conflicting results of trials. No firm evaluative statement. No citation bias present and cites all available RCTs. Cites Turpeinen et al. (1968), which is the *Finnish Mental Hospital* trial. Also cites Dayton et al. (1968), a RCT thought relevant only in primary prevention. |
| R19  [57] | Hulley, SB; Sherwin, R; Nestle, M; Lee, PR | It should come as no surprise, then, that no definitive randomized trial of preventing CHD by dietary change has yet been carried out. Of the studies that have been conducted, some have shown no benefit at all (Medical Research Council, 1968; Franz et al. 1975) while others have provided at best suggestive evidence for the efficacy of a fat-controlled diet (Miettinen et al. 1972; Dayton et al. 1969; Leren 1970)…In short, while we can conclude that reducing saturated fats and cholesterol in the diet does lower serum cholesterol levels, we have not yet established beyond doubt, probably because of technical barriers to conducting definitive research on the issue, that such diets will prevent heart disease. (p. 28) | Neutral | No evidence of citation bias – cites two of four secondary prevention RCTs. Also cites Frantz et al. (1975) and Dayton et al. (1969) – mixed primary and secondary RCTs believed relevant only to primary prevention, and Miettinen et al. (1972) – a non RCT. |
| R17  [47] | Simborg, DW | Most of the data in this regard has involved the use of hypocholesterolemic diets on men with a previous MI. Paul Leren’s (1966) controlled study of 412 men post-MI showed that there were less re-infarctions, less new cases of angina pectoris, and less deaths from MI in the 206 men treated by diet. The results became statistically significant after 3 years of diet in men less than 60 years of age. Other studies have had similar results in men with a previous MI [Morrison 1960; Bierenbaum et al. 1967)….One controlled study of men post MI failed to demonstrate a protective effect of a hypocholesterolemic diet (Research Committee 1965)...In a more recent study in London (Medical Research Council 1968), 393 post-MI male patients were randomized into a control group and hypocholesterolemic diet group. In this case, serum cholesterol dropped 22 per cent in the diet group and 6 per cent in the experimental group. In the follow up of 2–7 years, there was no significant difference in relapse rate between the two groups. The evidence of an effect of a hypocholesterolemic diet on the secondary prevention of CHD is *inconclusive*. (p. 520) | Neutral | Highlights conflicting results. No evidence of citation bias. Cites 3/4 available secondary prevention RCTs. Also cites two trials that are not RCTs – Morrison 1960; Bierenbaum et al. 1967) |
| R16  [53] | Shaper, AG | The earlier studies were based on the lowering of blood cholesterol by dietary means and the results were an unhappy mixture of encouragement and disappointment (Research Committee, 1965; Rose et al 1965; Leren, 1970; Medical Research Council 1968). (p. 467–468) | Neutral | Highlights conflicting results – no firm evaluative statement. No evidence of citation bias and cites all available RCTs. |
| R14  [51] | Jacobson, NL | There is some evidence (Leren, 1970; Pearce and Dayton 1971; Turpeinen et al. 1968) that a diet low in cholesterol and saturated fat not only will reduce serum cholesterol, but also will result in a small reduction in cardiovascular disease in man…On the other hand, Rose et al. (1965) observed no decrease in death rate, or in tissue damage in nonfatal events, in patients with ischemic heart disease (restricted blood flow due to narrowing of the arteries) when the dietary saturated fat was reduced and corn oil was added. In fact, there was some indication that the change to corn oil may have been harmful. (p. 144)...There is as yet no substantial evidence that mortality from coronary heart disease can be appreciably altered by qualitative dietary change alone. (p. 146) | Neutral | Highlights conflicting results. No evidence of citation bias. Utilises 2/4 available RCTs. Also cites the *Finnish Mental Hospital Study* and Dayton’s *LA Veterans Study* [however, the reference for this – Pearce and Dayton 1971 – actually refers to a paper examining the results of their dietary fat trial and the incidence of cancer. |
| R12  [60] | Stallones, RA | In three studies oil was added to the diet to increase the P/S ratio (Leren 1966; Rose et al 1965; Medical Research Council 1968), and in the fourth the experimental diet was low in fat (Research Committee 1965)…None of the studies in Great Britain offered a glimmer of hope, and the one in Norway has been variously interpreted. Leren (1966) concluded that his findings in Oslo supported the proposition that dietary modification protected study subjects under the age of 60 years from subsequent relapse. The Committee of the British Medical Research Council (1968) directing the Soya Oil Study compared their results with those of Leren and concluded that they were "remarkably similar"-i.e. both *negative.* Attention has been drawn to the fact that the most favorable results in Oslo I were observed in the category of acquired angina pectoris (although these differed little from those in the category of fatal myocardial infarction), and that the persons evaluating angina pectoris were not blinded to the assignment to the treated and control groups…Proponents of the diet-heart hypothesis have argued that all of these picky points are irrelevant, since an effective dietary regimen may have limited (or no) value in men who have already experienced severe ischemic heart disease. (p. 171) | Neutral | Highlights conflicting trial results – no firm evaluative statement. No citation bias present and cites all of the available RCTs. |
| R67  [48] | Zelis, RD; Mason, DT; Spann JF | Can one affect the course of vascular disease by altering the levels of serum cholesterol? Although population studies have tended to answer this question in the affirmative (Laren 1966; Dayton et al. 1968), the results of prospective studies have not been striking and there has been one study which did not show increased longevity with the treatment of hyperlipidemia (Medical Research Council 1968) | Neutral | Highlight conflicting results – no firm evaluative statement offered. No evidence of citation bias and utilisation of 2/4 RCTs. Also cites Dayton et al. (1968) – a mixed primary and secondary prevention RCT thought relevant only to primary prevention.  Note: This paper misspelled Leren as “Laren” and will not be detected on indexed citation searches due to this. |
| R10  [56] | Lewis, B | Earlier trials of secondary prevention of IHD were less consistent (Medical Research Council Research Committee 1968, Leren 1966), (p. 810) | Neutral | Highlights conflicting results – no firm evaluative statement. No evidence of citation bias and cites 2/4 available RCTs. |
| R62  [50] | Stormorken, H | The dietary trials have yielded conflicting results on the effect of lowering blood cholesterol. Three rather well-designed British studies were negative (Rose et al 1965; Research Committee 1965; Medical Research Council 1968), whereas there are various degrees of significant effect in other materials - well and less well designed, small and large - taken together (Leren 1966). (p. 10) | Neutral | Highlights conflicting results – no firm evaluative statement. No evidence of citation bias and utilises all available RCTs. |
| R65  [49] | Logue, B; Robinson P H | Leren (1966) has claimed reduction of the incidence of fatal and nonfatal myocardial infarction in patients on diets low in saturated fats and cholesterol. The study of the British Medical Research Council (1968) did not confirm such benefits. (p. 1133) | Neutral | Highlights conflicting results – no firm evaluative statement. No evidence of citation bias and cites 2/4 available RCTS. |
| **Supportive Reviews** | | | | |
| R61  [90] | Kannel, W | Conclusive evidence incriminating diet in atherogenesis in the free-living general population continues to be elusive. Further studies attempting to establish a connection between habitual dietary practices and either serum lipid values or coronary heart disease rates within general population samples have been largely unsuccessful (Kannel and Gordon 1970). Efforts to do so in special population subgroups have been more successful (Dayton et al. 1969; Miettinen et al. 1972; Leren 1970) (p. 31) | Supportive | While Kannel appears to stress the lack of conclusive evidence in this area, he references the *Framingham* study (1970), a large longitudinal cohort study as providing unsupportive/unsuccessful results. However, he goes on to say that other efforts have been more successful – of which he quotes three intervention trials – of which one is a secondary prevention RCT by Leren. He also cites Dayton et al. (1969) mixed prevention RCT and Mierttinen et al. *Finnish Mental Hospital* trial – a non RCT. Thus, this is an example of citation bias favouring supportive data – utilising 1/4 secondary prevention RCTs. |
| R60  [102] | Hornstra, G | Human prospective studies have also shown that diets enriched in linoleic acid are beneficial in the prevention of atherosclerosis and its complications (Leren 1970; Miettinen et al. 1972; Dayton et al. 1969) (p. 407) | Supportive | Citation bias favouring supportive trial – utilises 1/4 available secondary prevention RCTs. Also cites Dayton et al. (1969) mixed prevention RCT and Mierttinen et al. *Finnish Mental Hospital* trial – a non RCT |
| R59  [91] | Grundy, S M; Bilheimer, D; Blackburn, H; Brown, V; Kwiterovjch, P O; Mattson, F; Schonfe G; Wekdman, W H | Finally, two intervention trials have been carried out in Oslo, Norway (Leren 1966, Hjermann et al. 1981). The first, reported in 1966, was a secondary prevention trial using a low-cholesterol, highpolyunsaturated fat diet. The results suggested that use of this diet reduced recurrent rates of myocardial infarction. (p.182).  Although all the above studies [see comment box] were flawed in one or another aspect of experimental design, they were uniform in reporting a favorable trend toward decreased CHD risk with cholesterol-lowering diets. (p. 182) | Supportive | Citation bias for supportive trials and utilises 1/4 available secondary prevention RCTs. The author refers to Leren (1966) and a later multifactorial primary prevention trial (Hjermann et al. 1981). Also, not in quote is a summary of other non-relevant trials to secondary prevention (Rinzler 1968; Miettinen et al. 1972; Franz et al. 1975; Dayton et al. 1969). As the focus of this review was on primary prevention trials this is understandable; however, to include *only* the single secondary prevention RCT which came to a supportive result in this discussion is not. |
| R58  [82] | Christakis, G; Rathmann, D | It is evident that in these studies with middle-aged and elderly individuals, *diet modification decreased the incidence of coronary events and often decreased mortality ascribable to atherosclerotic disease* … None of the studies so far reported has been completely satisfactory with respect to design or execution. Accordingly, the results do not unequivocally prove, but strongly suggest, that modification of diet with respect to type and amount of fat is effective in the prevention of coronary heart disease…At best, the dietary change may modify the factors of risk to such a degree as to completely prevent a coronary episode, even in the presence of coronary atherosclerosis(p. 274) | Supportive | Clearly supportive. This statement comes after the reporting of the raw results of 9 secondary prevention trials (6 modified fat intervention trials: Nelson 1956, Hood 1965, Rose et al. 1965, Leren 1966, Bierenbaum et al. 1970, and Medical Research Council 1968); 3 restricted fat intervention trials: Lyon et al 1965, Morrison 1960, and Research Committee 1965). Of these, only four are RCTs.  No evidence of citation bias and full utilisation of available RCTs. |
| R57  [104] | Council of Scientific Affairs. AHA | Whether serum lipid reductions can influence the course of CHD in the human species has not been established conclusively. However, there have been a few clinical trials involving patients with known or suspected CHD in which cardiovascular complications seemed to be favorably influenced by plasma cholesterol reduction (Dayton et al 1968; Miettinen et al. 1972; Leren 1970). Thus, the evidence reinforces the concept that a strong relationship exists between lipid metabolism and atherogenesis (p. 1874) | Supportive | Bias for supportive trials – utilises 1/4 secondary prevention RCTs.  Also cites Dayton et al 1968 – a mixed RCT relevant only to primary prevention, and Miettinen et al. (1972) – a non RCT. |
| R55  [93] | Eaton, P R | Long term intervention studies of men in the ‘premature’ age with known coronary heart disease, have demonstrated that diet alone…may reduce progression of the disease…the 5 yr therapeutic intervention of Leren (1966)…and in New Jersey (Bierenbaum et al. 1970)… supported the concept of beneficial intervention in patients with known coronary disease based upon the incidence of new coronary events.(p. 131) | Supportive | Author cites two secondary prevention dietary trials (Leren, 1966; Bierenbaum et al. 1970). Of these, only Leren (1966) is a RCT. Here, we see citation bias favouring supportive trials and cites 1/4 available RCTs. |
| R54  [80] | Little, J A | The dietary trials by Leren (1996), Turpeinen et al. (1968), Dayton et al. (1968), and Christakis et al. (1966), suggest that altering the diet of healthy or coronary population groups lowers serum lipids, as long as the diet is maintained, and reduces the incidence of atherosclerotic complications. The British Trial (Medical Research Council 1968) did not achieve similar results. Malmros (107) critically reviewed these studies and concluded that the diet should be changed for the entire population…Thus it is the author’s opinion that both groups, the primary hyperlipoproteinemias and the so-called normal population, would likely benefit from a nationwide nutritional program which would significantly lower serum lipids throughout a lifetime. Even over a span of a few years Leren in Norway and Turpeinen et al. in Finland have shown that substituting polyunsaturated fats and oils for animal and dairy fats significantly lowered serum cholesterol and reduced morbidity from clinical atherosclerosis (p. 33–34) | Supportive | No evidence of citation bias – cites 2/4 available secondary prevention RCTs.  Also cites Turpeinen et al. (1968), Dayton et al. (1968), and Christakis et al. (1966) – see previous boxes for reason for exclusion. |
| R45  [105] | Furberg, C D; May, G S | In all three dietary trials (Research Committee 1965; Leren 1966; Medical Research Council 1968), a lower mortality was reported in the intervention group...in all three trials of low-fat diets a favorable trend was reported. Although a beneficial effect on survival has not been demonstrated, it seems prudent to recommend dietary intervention in post-myocardial infarction patients with elevated cholesterol levels. (p. 80–81) | Supportive | No evidence of citation bias – cited 3/4 secondary prevention RCTs. Erroneously suggests two of these studies (Medical Research Council 1968; Leren 1966) were low-fat trials - they were rather fat modified trials attempting to assess the impact of increasing PUFA while decreasing SFA. This meant that the control and intervention group had roughly equivalent fat intakes and <30% of calories. |
| R44  [79] | Rathmann, D M; Stockton, R J; Melnick, D; Stare, F | In the eight reports (Medical Research Council 1968; Leren 1966; Rose et al 1965; Hood et al. 1965; Nelson 1956, Bierenbaum et al. 1967; Christakis et al. 1966; Leren 1966; Turpeinen et al. 1968) which we have just outlined so sketchily, there has been no unanimity with respect to experimental design, choice of participants, extent of diet modification, measurement of adherence, or clinical criteria of coronary events…Notwithstanding the tremendous differences in designs of these studies results show that sufficiently motivated individuals can make this dietary change and that the net effect is a reduction in their risk of having a heart attack. (p. 358) | Supportive | No evidence of citation bias and utilises 3/4 secondary prevention trials. Although the exclusion of Research Committee (1965), in this case, seems appropriate as the authors focus on the effect of fat modification (replacing SFA with PUFA).  Also cites (Hood et al. 1965; Nelson 1956, Bierenbaum et al. 1967; Christakis et al. 1966 Turpeinen et al. 1968) all of which are not RCTs. |
| R43  [101] | Steinberg, D | …Three published studies suggesting that low-cholesterol, low-saturated fat diets both lower plasma cholesterol levels and reduce incidence of atherosclerotic complications (Leren, 1966; Dayton et al. 1969; Miettinen, et al. 1972). The number of subjects studied was less than optimal and none of the studies was free of flaws in experimental design and problems in interpretation. Nevertheless, the positive results must not be ignored. Thus, the "lipid hypothesis" is not without support from direct experimental testing in the ultimate arena-clinical trials. (p. 78) | Supportive | Citation bias favouring supportive trials – utilises only 1/4 available secondary prevention RCTs. Also cites the *Finnish Mental Hospital Study* – (Miettinen et al. 1972), which is not a RCT. Finally cites Dayton et al. (1969) mixed primary and secondary prevention RCT – relevant only to primary prevention. |
| R42  [103] | Grundy, S M | …attempts have been made to test the lipid hypothesis in field trials using both diets and drugs to lower plasma cholesterol levels. Indeed, several field trials strongly suggest that lowering plasma cholesterol will decrease clinical manifestations of atherosclerosis (Leren 1966; Miettinen et al. 1972; Dewar and Oliver 1971; Krasno and Kidera 1972) (p. 985) | Supportive | Citation bias favouring supportive trials – utilises 1/4 secondary prevention RCTs. Also cites Miettinen et al (1972) – a non RCT. Also references two drug trials (Dewar and Oliver 1971; Krasno and Kidera 1972). |
| R39  [96] | Hegsted, D M | …many dietary trials (Leren 1970; Dayton et al 1969; Miettinen et al 1972; Bierenbaum et al 1973) do show the situation is not that discouraging even for those of us who have already developed considerable atherosclerosis. Most of the studies in which serum cholesterol has been lowered by dietary means have shown a decrease in CHD rate. (p. 2000) | Supportive | Citation bias favouring supportive trials – utilises 1/4 secondary prevention RCTs.  Also cites Dayton et al. 1969; Miettinen et al. 1972; Bierenbaum et al. 1973) – see previous boxes for reason for exclusion. |
| R37  [88] | Glueck, ; Connor, WE | …in four dietary modification trials completed (Dayton et al 1969; Miettinen et al 1972; Leren 1966; Franz et al. 1975), there was significant lowering of plasma cholesterol, and suggestive but not unequivocal reduction in "hard" and "soft" end points for CHD. (p. 120–121) | Supportive | Citation bias favouring unsupportive trial – utilises 1/4 available RCTs.  Cites Dayton et al. 1969, Miettinen et al. 1972, Franz et al. 1975 – see previous boxes for reason for exclusion. |
| R35  [100] | Stamler, J | Thus, hypercholesterolemia…is one of few post-MI risk factors amenable to safe modification (by dietary means in the case of hypercholesterolemia). The inference is that a sizeable potential exists to reduce absolute risk for these very high risk patients. The encouraging data from two secondary prevention trials involving serum cholesterol control by dietary means lend support to this inference (Kallio et al. 1979; Leren 1966) (p. 437) | Supportive | Citation bias favouring supportive trials – utilised 1/4 secondary prevention trials. Cites Kallio (1979), which is a multifactorial intervention trial (lowering smoking, β blockers, and ‘nutrition’ education). |
| R32  [83] | Stamler, J | Recent data from the Coronary Drug Project indicate that serum cholesterol level remains predictive of risk of dying for men who have recovered from one or more myocardial infarctions in middle age. This new finding indicates that there is also a substantial rationale for treatment of hypercholesterolemia in patients with frank clinical coronary heart disease, for purposes of secondary prevention. In this regard, the Coronay Drug Project data are consistent with the results of at least one European study (Leren, 1966) (p. 23) | Supportive | Bias for supportive trials – utilises 1/4 secondary prevention trials. Also cites a drug intervention study. |
| R31  [94] | Steinberg, D | The question of whether correction of hyperlipidemia reduces risk is controversial. The weight of experimental and epidemiologic evidence is at least as good as - or actually better than - the evidence that correcting hyperglycemia reduces risk of chronic diabetic complications. Three studies (Leren, 1966; Dayton et al. 1969; Miettinen et al. 1972) present evidence to suggest that low-cholesterol, low-saturated fat diets both lower plasma cholesterol levels and reduce the incidence of atherosclerotic complications. The number of subjects studied was less than optimal and none of the studies was free of flaws in experimental design or problems in interpretation. Nevertheless, the positive results recorded must not be ignored. Thus, the "lipid hypothesis" is not without support from direct experimental testing in clinical trials. (p. 78) | Supportive | Citation bias favouring supportive trials – utilises 1/4 secondary prevention RCTs.  Also cites Dayton et al. (1969) and Miettinen et al. (1972) – see other boxes for reason for exclusion. |
| R30  [87] | Grande, F | Although none of the studies ([of which secondary prevention] Leren 1966) considered provides incontrovertible evidence of the value of the diet in preventing CHD, they clearly support the view that dietary manipulation designed to decrease the serum cholesterol level is useful in reducing the risk of CHD. (p. 1667) | Supportive | Bias for supportive trials – utilises 1/4 available secondary prevention RCTs. |
| R28  [78] | Malmros, H | Some ten dietary trials on survivors of myocardial infarction have been published: in most of them the results have been encouraging. Only two of the most extensive and best controlled trials will be discussed here, namely Leren’s (1966) investigation in Oslo and that of Morris and others in London (Medical Research Council 1968). The Oslo trial gave a positive result, while the London investigation produced no evidence for the assumption that the relapse-rate in myocardial infarction can be appreciably affected by diet… This difference in diet might explain why the results of the London group were not so favourable as those of the Oslo trial. Neither were the patients strictly comparable. In the London trial the patients had recently had their first infarction, on the average, within the previous 36 days. In the Oslo trial the corresponding period was 20 months. As known, the risk of a relapse is especially high during the first year after the infarction-i.e., before the diet would have had a chance to exert its effect. The Oslo series also included patients with a high blood-pressure, and the serum-cholesterol at the beginning of the trial was higher than in the London series. *Probably, dietary treatment is especially useful in the treatment of patients who have both hypercholesterolsemia and hypertension* (p. 481–482) | Supportive | No evidence of citation bias – cites 2/4 available secondary prevention RCTs. Classified as supportive on basis of last sentence. |
| R27  [99] | Hornstra, G | The value of epidemiological studies, however, is rather limited as they only detect associations but are not suitable to prove cause-and-effect relationships. For this latter purpose, prospective clinical trials are needed, several of which have now been completed, all indicating a favourable effect of a diet low in saturated fats and enriched in linoleic acid (Leren, 1966; Dayton et al. 1969; Miettinen et al. 1972). (p. 564–565) | Supportive | Citation bias favouring supportive trials – utilises 1/4 secondary prevention trials.  Also cites Dayton et al. (1969) and Miettinen et al. (1972) – see above boxes for reasons for exclusion. |
| R25  [98] | Hymowitz, N | …the success of several clinical trials, some with men before the development of heart disease (e.g., National Diet-Heart Study, 1968) and some with people who had already suffered a major coronary event (Leren, 1966), suggest that serum cholesterol modification is possible and practical for large numbers of people. (p. 41–42) | Supportive | Citation bias favouring supportive trials – utilises 1/4 secondary prevention trials. |
| R24  [92] | Hegsted, DM | Fortunately, most of the well-designed trials (Leren 1970; Miettinen et al. 1972; Dayton et al. 1969) have shown an amelioration of CHD after dietary modification which lowered the serum cholesterol. They indicate that advice to middle-aged men is not without benefit but, as expected, younger men have more to gain than older men…No responsible individual can conclude that further research is not necessary or that it will be unproductive. Research is the only source of new knowledge. Lack of knowledge, however, does not absolve the scientific community from making responsible decisions with the knowledge available. We will never know all that we should know. (p. 1507) | Supportive | Citation bias favouring supportive trials – utilises 1/4 secondary prevention trials.  Also cites Miettinen et al (1972) and Dayton et al (1969) – see above boxes for reason for exclusion. |
| R23  [89] | Abrahamson, JH; Hopp, C | Secondary prevention trials of lipid-lowering diets have not all shown favorable effects (Dayton et al. 1968), but on balance they indicate that such diets probably do yield small benefits. Most of these trials were conducted on middle-aged men. The limited evidence suggests that the efficacy of such diets decreases with increasing age. In a study in New Jersey which showed a reduced incidence of myocardial reinfarction, this effect was less striking among men aged 45–54 than among those aged 30–44 years (Bierenbaum et al. 1970). In a study in Oslo, a lipid-lowering diet produced a significant reduction of coronary relapses over a 5-year period among men aged 30–59 years, but a smaller and nonsignificant reduction among men aged 60–67 years (Leren 1966, 1970). (p. 37–38). | Supportive | Citation bias favouring supportive trials – utilises 1/4 secondary prevention RCTs.  Also cites Dayton et al. (1968), Bierenbaum et al. (1970) – see above boxes for reason for exclusion. |
| R21  [86] | Renaud, S | An additional evidence of the role of dietary fat in coronary thrombosis can be obtained from the prevention studies by dietary modifications. Several reports have shown that by changing the type of fat and reducing the amount of calories supplied by fats in the diet, the incidence of CHD can be significantly reduced in a population initially free from CHD clinical manifestations (Christakis et al. 1966) or in survivors of myocardial infarction (Leren 1966) (p. 31) | Supportive | Citation bias favouring supportive trials – cites 1/4 secondary prevention RCTs.  Also cites Christakis et al 1966 – a primary prevention non RCT. |
| R18  [95] | Glueck, CJ; Mattson, F; Bierman, E | A second discordant area has been the lack of unequivocal, uniform reduction in “hard” and “soft” end points in populations in which diets have been modified or where lipid-loweruing drugs have been given. In four dietary-modification trials completed (Dayton et al. 1969; Miettinen et al. 1972; Leren 1966; Franz et al. 1975), there was a significant lowering of plasma cholesterol, and suggestive, but not unequivocal, reduction in hard and soft end points for CHD. (p. 1472) | Supportive | Citation bias favouring supportive trials – cites 1/4 secondary prevention RCTs.  Also cites Dayton et al. 1969; Miettinen et al. 1972; Leren 1966; Franz et al. 1975 |
| R11  [85] | Mcbean, LD; Spechmann, EW | Thus it is emphasized that diet throughout the life of an individual, from infancy through adulthood, be lowered in saturated fatty acids and cholesterol and increased in polyunsaturated fatty acids in an attempt to prevent the development of elevated blood cholesterol levels (hypercholesterolemia) and hence atherosclerosis. Other investigators (Leren 1970; Dayton et al. 1968) have shown that in certain selected segments of the population dietary changes in cholesterol and/or saturated fatty acids have apparently decreased the incidence of heart disease. Dayton et al. and Leren have reported that a cholesterol-lowering diet fed to older men resulted in a decreased occurrence of myocardial infarction. (p. 837) | Supportive | Citation bias favouring supportive trial – cites only 1/4 available secondary prevention RCTs. |
| R63  [97] | Nora, J J | The specific influence of diet and other therapies in lowering cholesterol levels and in preventing coronary disease has been the subject of considerable debate. The interpretation of various studies is strongly subject to the perspective (bias?) of the interpreter. Our bias is that the data (Turpeinen et al. 1979; Leren 1970) support the idea that lowering cholesterol concentration by diet or medication reduces morbidity and mortality from IHD. It has been shown in a number of studies that the higher the level of cholesterol the higher the risk, and conversely (p. 710) | Supportive | Citation bias favour supportive trial – utilises 1/4 secondary prevention RCTs. Also cites *the Finnish Mental Hospital Study* - a non RCT. |
| R64  [84] | Anderson, J T; Grande, F; Keys, A | Serval studies involved dietary treatment in the secondary prevention of coronary heart disease. Although most of the earlier reports showed a favourable effect of diet, they have been ignored or criticized because of faulty statistical design…and so forth. A more recent study was conducted in Oslo, Norway, by Leren (1966)...During the five years there were significantly fewer recurrences of infarctions (*P* = 0.02) and death from infarctions (*P* = 0.03) among the dieters compared with the control group...Although none of these studies provides incontrovertiable evidence of the value of the diet in prevention coronary heart disesae, collectively they support the theory that dietary manipulation designed to decrease serum cholesterol...is useful in reducing the risk of coronary heart disease. (pp. 139–40) | Supportive | Citation bias favour supportive trial – utilises 1/4 secondary prevention RCTs. |
| R66  [81] | Vergroesen, AJ | Clinical trials generally confirmed the beneficial effect on ischaemic heart disease of an increased intake of linoleic acid and a decreased intake of saturated fatty acids and cholesterol (Turpeinen et al. 1968; Dayton et al. 1969; Leren, 1970) | Supportive | Citation bias favouring supportive trials – utilises 1/4 secondary prevention RCTs. |

**References cited in quotation data:**

To avoid confusion with references referenced in the manuscript, which are associated with an integer [*-*] label, the referenced articles in these quotation data are provided in alphabetical order below:

Bierenbaum M. Modified-fat dietary management of the young male with coronary disease. A five-year report. *JAMA.* 1967; 202: 1119-1123.

Bierenbaum ML, Fleischman AI, Green DP, Raichelson RI, Hayton T, Watson PB et al. The 5-year experience of modified fat diets on younger men with coronary heart disease. *Circulation.* 1970; 42: 943-952.

Bierenbaum M, Fleischman AI, Raichelson RI, Hayton T, Watson PB. Ten-year experience of modified-fat diets on younger men with coronary heart-disease. *Lancet.* 1973; 301: 1404-1407.

Christakis G, Rinzler SH, Archer M, Winslow G, Jampel S, Stephenson J et al. The anti-coronary club. A dietary approach to the prevention of coronary heart disease - a seven-year report. *Am J Public Health Nations Health*. 1966; 56: 299-314.

Dayton S, Pearce ML, Hashomoto S, Dixon WJ, Tomiyasu U. A controlled clinical trial of a diet high in unsaturated fat in preventing complications of atherosclerosis. *Circulation.* 1969; 40: II-1–II-63.

Dayton S, Pearce ML. Prevention of coronary heart disease and other complications of atherosclerosis by modified diet. *Am J Med*. 1964; 46:751.

Dayton S, Pearce, ML, Goldman H, Harnish A, Plotkin D, Shickman M et al. Controlled trial of a diet high in unsaturated fat for prevention of atherosclerotic complications. *Lancet*. 1968; 2: 1060-1062

Dewar HA, Oliver MF. Secondary prevention trials using clofibrate: a joint commentary on the Newcastle and Scottish trials. *Br Med J*. 1971; 4: 784-786.

Frantz ID, Dawson EA, Kuba K, Brewer ER, Gatewood LC, Bartsch GE. The Minnesota coronary survey: Effect of diet on cardiovascular events and deaths. American Heart Association Scientific Proceedings. *Circulation*. 1975; 41 (Suppl. 2): 2-4.

Hansen PF, Geill T, Lund E. Dietary fats and thrombosis. *Lancet.* 1962; 2:1193-1194.

Hjermann I, Velve Byre K, Holme I, Leren P. Effect of diet and smoking intervention on the incidence of coronary heart disease: report from the Oslo Study Group of a randomised trial in healthy men. *Lancet*. 1981; 318: 1303-1310.

Hood B, Sanne H, Örndahl G, AhlstÖm M, Welin G. Long-term prognosis in essential hypercholesterolemia. *Actu Med Scand.* 1965; 178: 161-173.

Kallio V, Hämäläinen H, Hakkila J, Luurila OJ. Reduction in sudden deaths by a multifactorial intervention programme after acute myocardial infarction. *Lancet.* 1979; 314: 1091-1094.

Kannel WB, Gordon T, eds. The Framingham Study: diet and regulation of serum cholesterol, Section 24. In: *The Framingham Study: An Epidemiological Investigation of Cardiovascular Disease*. Washington, DC: U.S. Government Printing Office, 1970.

Krasno LR, Kidera GJ. Clofibrate in coronary heart disease: effect on morbidity and mortality. *JAMA*. 1972; 219: 845-851.

Leren P. The effect of plasma-cholesterol-lowering diet in male survivors of myocardial infarction. A controlled clinical trial. *Acta Med Scand Suppl.* 1966; 466: 1-92.

Leren P. The Oslo diet-heart study. Eleven-year report. *Circulation.* 1970; 42:935-942.

Lyon TP, Yankley A, Gofman JW, Strisower B. Lipoproteins and diet in coronary heart disease. *Calif Med.* 1956; 84: 325-328.

Medical Research Council. Controlled trial of soya-bean oil in myocardial infarction: Report of a research committee to the Medical Research Council. *Lancet.* 1968; 292: 693-700.

Miettinen M, Turpeinen O, Karvonen MJ, Elosuo R, Paavilainen E. Effect of cholesterol-lowering diet on mortality from coronary heart-disease and other causes. A twelve-year clinical trial in men and women. *Lancet*. 1972; 2: 835-838.

Morrison LM. Diet in coronary atherosclerosis. *J Am Med Assoc*. 1960; 173:884-888.

National Diet-Heart Study Research Group. The National Diet-Heart Study Final Report. *Circulation*. 1968; **37**(3 Suppl.): I1-428.

Nelson AM. Treatment of atherosclerosis by diet. I. Results in patients followed from 36 to 72 months. *Northwest Med*. 1965; 55: 643-649.

Pearce ML, Dayton S. Incidence of cancer in men on a diet high on polyunsaturated fat. *Lancet*. 1971; 1: 464-467.

Ramsden CE, Zamora D, Majchrzak-Hong S, Faurot KR, Broste SK, Frantz RP et al. Re-evaluation of the traditional diet-heart hypothesis: analysis of recovered data from Minnesota Coronary Experiment (1968-73). *BMJ.* 2016; 353: i1246. DOI: 10.1136/bmj.i1246.

Research Committee. Low-fat diet in myocardial infarction: a controlled trial. *Lancet.* 1965; 2: 501–504.

Rinzler SH. Primary prevention of coronary heart disease by diet. *Bull N Y Acad Med.* 1968; 44: 936-949.

Rose GA, Thomson WB, Williams RT. Corn oil in treatment of ischaemic heart disease. *BMJ.* 1965; 1: 1531-1533.

Turpeinen O, Miettinen M, Karvonen MJ, Roine P, Pekkarinen M, Lehtosuo EJ et al. Dietary prevention of coronary heart disease: Long-term experiment I. Observations on male subjects. *Am J Clin Nutr.* 1968; 21: 255-276.

Turpeinen O, Karvonen MJ, Pekkarinen M, Miettinen M, Elosuo R, Paavilainen E. Dietary prevention of coronary heart disease: the Finnish Mental Hospital Study. *Int J Epidemiol.* 1979; 8: 99-118.
