# Supplementary material for: A network analysis of the propagation of evidence regarding the effectiveness of fat-controlled diets in the secondary prevention of coronary heart disease (CHD): Selective citation in reviews
Source: PLoS One. 2018 May 24;13(5):e0197716. doi: 10.1371/journal.pone.0197716 (PMC5968408; doi:10.1371/journal.pone.0197716)
Supplement: S6 Table — (DOCX) [file pone.0197716.s006.docx]

**S6 Table. Attribute data on all studies (vertices) included in this study.**

| **Vertex Tag** | **Reference** | **Classification** |
| --- | --- | --- |
| RCT1 | 9 | Unsupportive |
| RCT2 | 8 | Unsupportive |
| RCT4 | 11 | Unsupportive |
| R1 | 75 | Unsupportive |
| R2 | 76 | Unsupportive |
| R3 | 74 | Unsupportive |
| R4 | 65 | Unsupportive |
| R5 | 66 | Unsupportive |
| R6 | 69 | Unsupportive |
| R7 | 73 | Unsupportive |
| R8 | 59 | Neutral |
| R9 | 54 | Neutral |
| R10 | 56 | Neutral |
| R11 | 85 | Supportive |
| R12 | 60 | Neutral |
| R13 | 71 | Unsupportive |
| R14 | 51 | Neutral |
| R16 | 53 | Neutral |
| R17 | 47 | Neutral |
| R18 | 95 | Supportive |
| R19 | 57 | Neutral |
| R20 | 45 | Neutral |
| RCT3 | 10 | Supportive |
| R21 | 86 | Supportive |
| R22 | 63 | Unsupportive |
| R23 | 89 | Supportive |
| R24 | 92 | Supportive |
| R25 | 98 | Supportive |
| R26 | 72 | Unsupportive |
| R27 | 99 | Supportive |
| R28 | 78 | Supportive |
| R29 | 68 | Unsupportive |
| R30 | 87 | Supportive |
| R31 | 94 | Supportive |
| R32 | 83 | Supportive |
| R33 | 44 | Neutral |
| R34 | 61 | Unsupportive |
| R35 | 100 | Supportive |
| R37 | 88 | Supportive |
| R38 | 64 | Unsupportive |
| R39 | 96 | Supportive |
| R40 | 62 | Unsupportive |
| R41 | 55 | Neutral |
| R42 | 103 | Supportive |
| R43 | 101 | Supportive |
| R44 | 79 | Supportive |
| R45 | 105 | Supportive |
| R47 | 77 | Unsupportive |
| R48 | 70 | Unsupportive |
| R50 | 58 | Neutral |
| R51 | 52 | Neutral |
| R52 | 46 | Neutral |
| R53 | 67 | Unsupportive |
| R54 | 80 | Supportive |
| R55 | 93 | Supportive |
| R57 | 104 | Supportive |
| R58 | 82 | Supportive |
| R59 | 91 | Supportive |
| R60 | 102 | Supportive |
| R61 | 90 | Supportive |
| R62 | 50 | Neutral |
| R63 | 97 | Supportive |
| R64 | 84 | Supportive |
| R65 | 49 | Neutral |
| R66 | 81 | Supportive |
| R67 | 48 | Neutral |
