# Supplementary material for: A network analysis of the propagation of evidence regarding the effectiveness of fat-controlled diets in the secondary prevention of coronary heart disease (CHD): Selective citation in reviews
Source: PLoS One. 2018 May 24;13(5):e0197716. doi: 10.1371/journal.pone.0197716 (PMC5968408; doi:10.1371/journal.pone.0197716)
Supplement: S7 Table — (DOCX) [file pone.0197716.s007.docx]

**S7 Table. Edge data on all citations between reviews and RCTs included in this study.**

| **Citing** | **Cited** |
| --- | --- |
| R62 | RCT1 |
| R58 | RCT1 |
| R52 | RCT1 |
| R48 | RCT1 |
| R47 | RCT1 |
| R45 | RCT1 |
| R34 | RCT1 |
| R33 | RCT1 |
| R29 | RCT1 |
| R6 | RCT1 |
| R20 | RCT1 |
| R17 | RCT1 |
| R16 | RCT1 |
| R1 | RCT1 |
| R13 | RCT1 |
| R12 | RCT1 |
| R22 | RCT1 |
| R62 | RCT2 |
| R58 | RCT2 |
| R52 | RCT2 |
| R47 | RCT2 |
| R44 | RCT2 |
| R38 | RCT2 |
| R34 | RCT2 |
| R33 | RCT2 |
| R20 | RCT2 |
| R16 | RCT2 |
| R4 | RCT2 |
| R1 | RCT2 |
| R14 | RCT2 |
| R12 | RCT2 |
| R22 | RCT2 |
| R67 | RCT4 |
| R65 | RCT4 |
| R62 | RCT4 |
| R58 | RCT4 |
| R54 | RCT4 |
| R52 | RCT4 |
| R51 | RCT4 |
| R50 | RCT4 |
| R48 | RCT4 |
| R45 | RCT4 |
| R44 | RCT4 |
| R40 | RCT4 |
| R38 | RCT4 |
| R34 | RCT4 |
| R33 | RCT4 |
| R10 | RCT4 |
| R9 | RCT4 |
| R8 | RCT4 |
| R7 | RCT4 |
| R29 | RCT4 |
| R6 | RCT4 |
| R20 | RCT4 |
| R2 | RCT4 |
| R17 | RCT4 |
| R16 | RCT4 |
| R4 | RCT4 |
| R28 | RCT4 |
| R1 | RCT4 |
| R13 | RCT4 |
| R12 | RCT4 |
| R3 | RCT4 |
| R19 | RCT4 |
| R1 | RCT3 |
| R2 | RCT3 |
| R3 | RCT3 |
| R4 | RCT3 |
| R5 | RCT3 |
| R7 | RCT3 |
| R8 | RCT3 |
| R9 | RCT3 |
| R10 | RCT3 |
| R11 | RCT3 |
| R12 | RCT3 |
| R14 | RCT3 |
| R16 | RCT3 |
| R17 | RCT3 |
| R18 | RCT3 |
| R19 | RCT3 |
| R20 | RCT3 |
| R67 | RCT3 |
| R66 | RCT3 |
| R65 | RCT3 |
| R64 | RCT3 |
| R63 | RCT3 |
| R62 | RCT3 |
| R61 | RCT3 |
| R60 | RCT3 |
| R59 | RCT3 |
| R58 | RCT3 |
| R57 | RCT3 |
| R55 | RCT3 |
| R54 | RCT3 |
| R53 | RCT3 |
| R52 | RCT3 |
| R51 | RCT3 |
| R50 | RCT3 |
| R47 | RCT3 |
| R45 | RCT3 |
| R44 | RCT3 |
| R43 | RCT3 |
| R42 | RCT3 |
| R41 | RCT3 |
| R40 | RCT3 |
| R39 | RCT3 |
| R38 | RCT3 |
| R37 | RCT3 |
| R32 | RCT3 |
| R34 | RCT3 |
| R33 | RCT3 |
| R26 | RCT3 |
| R25 | RCT3 |
| R24 | RCT3 |
| R31 | RCT3 |
| R35 | RCT3 |
| R30 | RCT3 |
| R28 | RCT3 |
| R27 | RCT3 |
| R23 | RCT3 |
| R21 | RCT3 |
